# Assembly-Driven Community Genomics of a Hypersaline Microbial Ecosystem

**DOI:** 10.1371/journal.pone.0061692

**Published:** 2013-04-18

**Authors:** Sheila Podell, Juan A. Ugalde, Priya Narasingarao, Jillian F. Banfield, Karla B. Heidelberg, Eric E. Allen

**Affiliations:** 1 Marine Biology Research Division, Scripps Institution of Oceanography, University of California San Diego, La Jolla, California, United States of America; 2 Department of Earth and Planetary Sciences, University of California, Berkeley, California, United States of America; 3 Department of Environmental Science, Policy, and Management, University of California, Berkeley, California, United States of America; 4 Department of Biological Sciences, University of Southern California, Los Angeles, California, United States of America; 5 Division of Biological Sciences, University of California San Diego, La Jolla, California, United States of America; Missouri University of Science and Technology, United States of America

## Abstract

Microbial populations inhabiting a natural hypersaline lake ecosystem in Lake Tyrrell, Victoria, Australia, have been characterized using deep metagenomic sampling, iterative *de novo* assembly, and multidimensional phylogenetic binning. Composite genomes representing habitat-specific microbial populations were reconstructed for eleven different archaea and one bacterium, comprising between 0.6 and 14.1% of the planktonic community. Eight of the eleven archaeal genomes were from microbial species without previously cultured representatives. These new genomes provide habitat-specific reference sequences enabling detailed, lineage-specific compartmentalization of predicted functional capabilities and cellular properties associated with both dominant and less abundant community members, including organisms previously known only by their 16S rRNA sequences. Together, these data provide a comprehensive, culture-independent genomic blueprint for ecosystem-wide analysis of protein functions, population structure, and lifestyles of co-existing, co-evolving microbial groups within the same natural habitat. The “assembly-driven” community genomic approach demonstrated in this study advances our ability to push beyond single gene investigations, and promotes genome-scale reconstructions as a tangible goal in the quest to define the metabolic, ecological, and evolutionary dynamics that underpin environmental microbial diversity.

## Introduction

Microbial diversity studies based on 16S rRNA gene amplification have identified large numbers of uncultured, uncharacterized organisms whose metabolic capabilities, lifestyle strategies, and ecosystem contributions remain largely unknown. Conversely, the subset of cultured microbial species from any particular habitat often fails to include even some of the most abundant members of the community. Efforts to bring these unknown organisms into laboratory culture are confounded by our limited understanding of the metabolic specializations of environmental microorganisms, the interdependencies of intra-/inter-species interactions, and the physicochemical conditions that promote or diminish microbial survival and population structure in natural environments.

Direct metagenomic sequencing of environmental samples can potentially provide functional information missing from 16S rRNA gene surveys and circumvent the constrained diversity found in representative cultured isolates. Composite genomes have been assembled from several environmental data sets [1], [2], [3], [4], [5], [6], however comprehensive characterization of the genetic diversity of most naturally occurring microbial communities remains a significant challenge. Environmental sampling of predicted metabolic functions as a simple “bag of genes” via metagenomic read-based analysis cannot fully capture the genetic and metabolic potential of individual populations, and may overlook the significance of community-wide processes involving cooperative interactions between multiple species [7], [8], [9].

Reference genomes from cultured isolates and/or single-cell projects can greatly assist in taxonomic assignment of genes encoded on short metagenomic DNA fragments. However, with the recent exception of the human microbiome project [10], the time, effort, and expense required to develop reference resources of sufficient breadth to adequately represent the full diversity of most ecosystems using these methods are currently prohibitive, and the vast majority of environmentally identified species remain uncharacterized.

The issue of inadequate database representation is particularly relevant for microbial communities in extreme hypersaline aquatic environments, which are dominated by archaeal populations [11]. These environments provide an attractive model for studying microbial ecology, because the demands of surviving such extreme conditions limit taxonomic diversity, yet cell densities frequently exceed 10^7^–10^8^ per mL [12]. The aquatic milieu allows convenient large-scale sampling and fractionation of discrete populations in particular size ranges, simplifying many types of analysis. These ecosystems have been well-studied historically using culture dependent-methods, 16S rRNA gene surveys and, more recently, single-cell genomics and metagenomics (reviewed in [11]). Despite these advances, the number of available sequenced genomes relevant to microbial communities in this specific habitat remains very small, and is not representative of the *in situ* diversity present in a natural microbial assemblage.

The extreme hypersaline habitat of Lake Tyrrell, Australia has recently been used to demonstrate the utility of *de novo* metagenomic assembly for characterizing organisms previously known only by their 16S rRNA gene sequences, including representatives of a globally distributed new class of Archaea, the Nanohaloarchaea [13], [14]. In the current study, we extend this previous work, combining cell size-fractionated sample collection, deep metagenomic sequencing, multidimensional phylogenetic binning, and iterative *de novo* assembly to reconstruct ten additional population genomes. These new genomes provide a comprehensive, culture-independent genomic blueprint for ecosystem-wide analysis of protein functions, population structure, and lifestyles linked to specific microbial strains co-existing and co-evolving within the same natural habitat.

## Materials and Methods

### Sample Collection, Library Construction and Sequencing

Surface water samples collected from Lake Tyrrell, Victoria, Australia at 0.3 m depth were passed through filters of decreasing porosities (20 µm>3 µm>0.8 µm>0.1 µm) to obtain fractions enriched by cellular size [13]. Physical properties of the collection site are summarized in **Table S1**. Sanger sequencing libraries were constructed at the J. Craig Venter Institute using DNA from 0.8 µm and 0.1 µm filters [15], and sequenced using both paired-end Sanger sequencing and Roche 454 Titanium pyrosequencing (**Table S2**). 16S rRNA gene clone libraries were constructed from the same DNA samples used for sequencing, using archaeal primer sequences Arc21F (5′-TTCCGGTTGATCCTGCCGGA-3′) and Arc529R (5′-ACCGCGGCKGCTGGC-3′) and bacterial primer sequences 27F (5′-AGAGTTTGATCCTGGCTCAG-3′) and 1391R (5′-GACGGGCRGTGWGTRCA-3′) [16].

### Lake Tyrrell Metagenome Assembly

Assemblies were performed using Celera Assembler software version 5.4 [17]. Read sizes, library sources, and the assembled positions of reads in contigs and scaffolds were extracted from the Celera Assembler ACE output file into a local MySQL database using custom perl scripts. Numbers of scaffold nucleotides, percentages of reads obtained from different libraries, and local coverage depth for specific scaffold subregions were calculated from SQL database queries.

**Figure S1** summarizes the bioinformatic assembly pipeline. All trimmed Sanger reads were combined into a composite pool for initial assembly. Scaffolds from this assembly were classified into groups using the phylogenetic binning procedures described below, then used to construct a custom reference library for PhymmBL version 3.2 [18], to assign unassembled 454 Titanium reads to taxonomic bins.

After an initial composite assembly of total community DNA, iterative rounds of *de novo* assembly were performed on taxonomic subgroups identified by scaffolds sharing common signatures based on multiple independent properties, to optimize assembly fidelity for each group individually. Each taxonomic subgroup was assembled independently using a previously described subtractive enrichment strategy based on iterative scaffold binning [13]. Scaffolds were re-binned and subsequently deconstructed into their component reads after each assembly iteration. Reads associated with scaffolds having properties characteristic of a subgroup other than the one currently being targeted were removed prior to the next round of assembly. To avoid over-pruning, singletons and reads associated with unclassified scaffolds were retained in successive rounds of assembly.

Taxonomic binning, subtractive enrichment, read deconstruction, and re-assembly steps were repeated for each taxonomic subgroup until no misassemblies were detected and no improvement was observed in completeness of conserved marker genes, maximum contig length, number and size of scaffold gaps, or uniformity of binning parameters for scaffolds >50 Kb. Assembly quality was confirmed by visual inspection using Hawkeye [19] to assess mate-pair consistency and read depth uniformity.

Archaeal genome assembly completeness was evaluated based on 53 transcription, translation, and replication genes nearly universally conserved in Archaea [20], [21], [22]. Bacterial draft genome completeness was assessed using the Core Gene Evaluation Script developed for the Human Microbiome Project [23]. Metagenomic sequence data has been deposited at DDBJ/EMBL/GenBank under the accession APHM00000000, NCBI BioProject number PRJNA59457. Assembled genome sequences have been deposited in the JGI-Integrated Microbial Genome resource [24] under taxon-oid numbers 2502082092 (J07HX64), 2506783034 (J07HB67), 2512875005 (J07HQW1), 2512875006 (J07HQW2), 2512875007 (J07HN4), 2512875008 (J07HN6), 2512875009 (J07HQX50), 2512875010 (J07HX5), 2512875011 (J07HR59), and 2513020022 (J07SB67).

### Phylogenetic Binning and Scaffold Annotation

Raw metagenomic reads and assembled scaffolds containing 16S rRNA gene sequences were identified by BLASTN search against the GreenGenes reference database [25], requiring a minimum alignment length of 200 nucleotides and e-value of 1e-7 or better. Scaffold genes were predicted and annotated using the Integrated Microbial Genomes Expert Review (IMG/ER and IMG/MER) systems [24]. Averaged amino acid frequencies for all predicted proteins on each scaffold were calculated using a custom perl script. Taxonomic associations of predicted protein matches to GenBank nr reference sequences were tallied using DarkHorse version 1.4 [26].

Non-metric multidimensional scaling (MDS) analysis was performed on scaffolds of 5000 nucleotides or longer containing <50% gap residues using Primer version 6.1.2 [27]. Scaffold input properties included nucleotide percent G+C; read depth; percent of reads from 0.1 µm filters; percentages of lysine, arginine, threonine, glutamic acid, aspartic acid, alanine, valine and isoleucine in predicted proteins; and percent of proteins with DarkHorse-filtered best matches to Eukaryota, Bacteria, Viruses, Nanohaloarchaea, and the genera *Haloquadratum*, *Halorabdus*, *Haloarcula*, *Halorubrum*, *Haloferax*, *Halogeometricum*, and *Salinibacter*. Scaffolds sharing a common signature based on these metrics were placed in the same taxonomic bin.

Unassigned scaffolds were searched against Lake Tyrrell-specific genome assemblies using BLASTN to identify potential variant sequences associated with strain level heterogeneity present in the natural population but not captured by targeted *de novo* assembly. Unassigned scaffolds matching a composite reference genome at 85% or higher average nucleotide identity (ANI) over >40% of their length were classified in the same “population group” as the matched genome [28]. Scaffolds matching at 95% or higher ANI were assigned to the same species. Total numbers of nucleotides for binned scaffolds in each population group, including species-level classifications, were calculated using SQL queries from assembly-specific MySQL databases, and converted to a proportional treemap graph using the TreeMap package in R, version 2.14.1 [29].

### Construction of Phylogenetic Trees

The Greengenes alignment tool NAST [25] was used to construct a reference alignment of 16S rRNA genes from assembled scaffolds, cultured isolate reference genomes, and closely related environmental sequences. Maximum likelihood reference trees were constructed using RaxML version 7.2.7 [30] and FastTree version 2.1.1 [31]. Partial 16S rRNA gene sequences from unamplified metagenomic reads and Lake Tyrrell PCR amplified clone libraries were inserted into reference trees using pplacer version 1.1 [32] and visualized using Archaeopteryx version 0.968 [33]. Amplified 16S rRNA sequences from Lake Tyrrell community DNA have been submitted to NCBI under accession numbers JX880413–JX81179 (archaeal) and JX881180–JX885105 (bacterial).

### Clustering of Predicted Proteins

Predicted proteins were clustered into families using an unsupervised Markov Clustering algorithm (MCL software version 10–201), with BLASTP e-value cutoff 1e-5 and inflation parameter setting 1.4 [34]. Protein family diversity was estimated using MOTHUR version 1.23.1 [35]. Assembled genomes were clustered together based on their profiles of shared protein families using the modularity analysis function of Gephi, version 0.8.1 [36].

## Results

### Community Sequence Assembly

**Figure 1:**
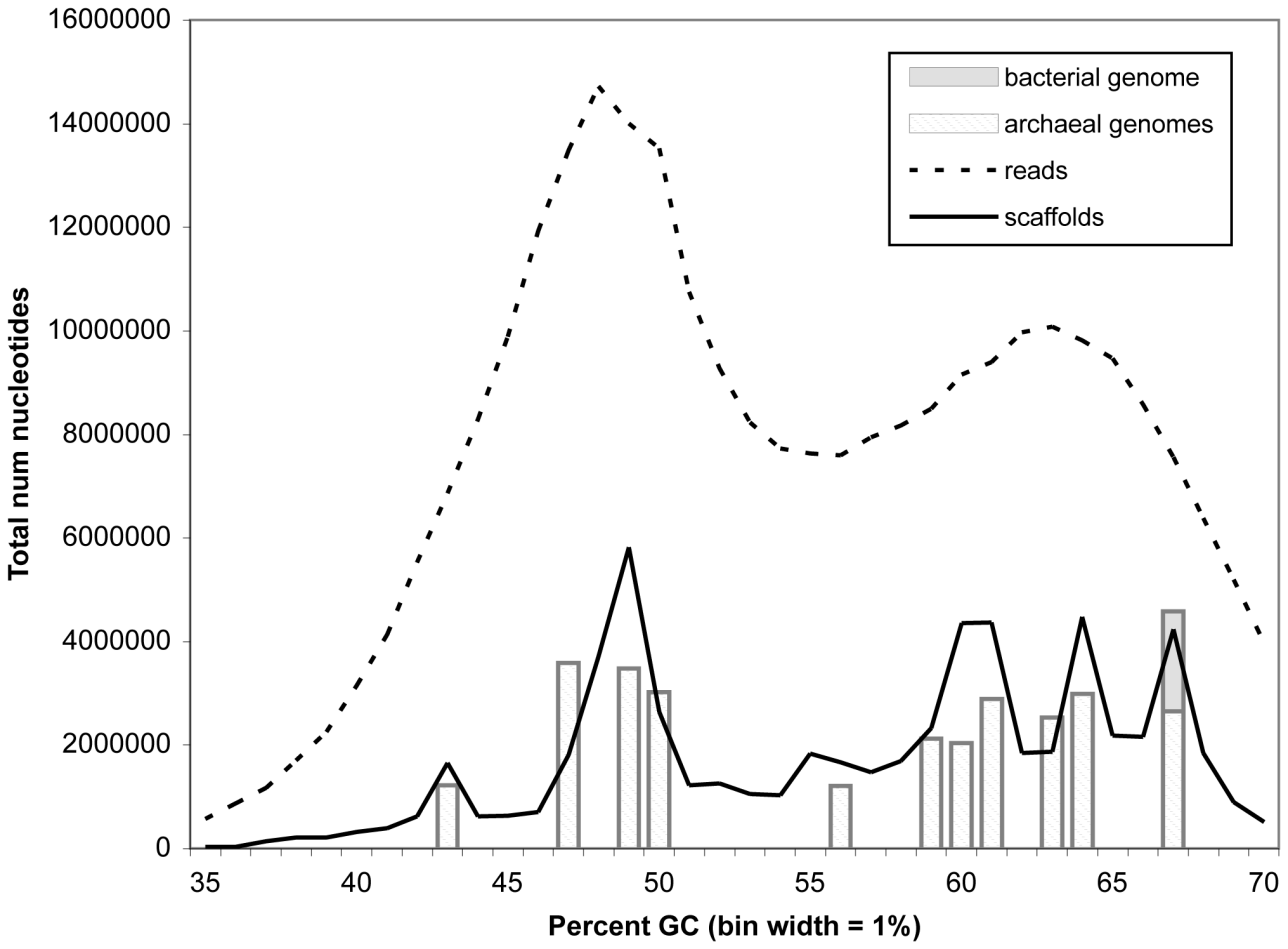
Length-weighted %G+C nucleotide composition of unassembled reads, assembled scaffolds, and composite population genomes. Genomes were constructed by targeted assembly of scaffolds with a uniform signature of phylogenetic binning properties, as described in Materials and Methods. Genome names, percent G+C, and other general properties of assembled genomes are shown in Table 1.

Metagenomic sequence assembly effectiveness for combined Sanger libraries was assessed statistically (**Table S3**), and visualized by comparing histograms of nucleotide composition (percent G+C) for unassembled reads versus assembled scaffolds and population genomes (**Figure 1**). Raw metagenomic sequencing reads prior to assembly have a broad, biphasic nucleotide distribution, reflecting their heterogeneous origin. The percent G+C distribution of assembled scaffolds is more tightly focused into discrete peaks because the assembly process consolidates multiple overlapping reads into longer, consensus sequences with uniform properties. The length-weighted nucleotide distribution for scaffolds thus reveals overall patterns that are hidden by random noise in the shorter read sequences.

Because the percent G+C content of individual microorganisms tends to be relatively uniform when averaged over long stretches of DNA, consolidated scaffold peaks in a length-weighted G+C histogram like **Figure 1** are useful in surveying diversity of dominant microbial populations within a mixed community. Prominent scaffold peaks at 43, 49, 56, 60–62, 64, and 67% GC suggested that the Lake Tyrrell microbial community contains at least 6 different abundant genomic populations. This observation was confirmed by the reconstruction of one or more composite genomes from each major peak (**Table 1**), including multiple archaeal populations with similar G+C compositions within broader peaks at 47–50%, 59–61%, and 63–64% G+C, and both archaeal and bacterial populations within the 67% G+C peak.

**Table 1:** Consensus population genome properties.

Genome name	Length (nt)	G+C pct	num scf	rRNA operons	tRNAs	predicted CDS	pct complete marker genes§
*Haloquadratum walsbyi* str J07HQW1	3,594,539	47	1	2	47	3,584	100
*Haloquadratum walsbyi* str J07HQW2	3,475,501	49	1	2	52	3,856	98
*Haloquadratum* sp. J07HQX50	3,019,909	50	2	1(2)*	39	2,872	91
*Nanosalinarum* sp. J07AB56	1,215,802	56	3	1	38	1,454	100
*Nanosalina* sp. J07AB43	1,227,157	43	7	1	59	1,739	83
*Halonotius* sp. J07HN4	2,888,659	61	2	1	52	3,230	100
*Halonotius* sp. J07HN6	2,529,000	63	6	1	47	2,914	100
uncultured archaeon sp. J07HX64	2,982,938	64	1	1	43	3,095	92
uncultured archaeon sp. J07HX5	2,040,945	60	1	1(2)*	24	2,139	53
*Halobaculum* sp. J07HB67	2,649,547	67	3	1	37	2,707	94
*Halorubrum* sp. J07HR59	2,120,805	59	7	1(3)*	26	1,841	83
*Salinibacter* sp. J07SB67	1,931,021	67	443	nd	13	1,641	39

§ Marker gene detection details are shown in Table S6.

* Parenthetical values indicate cases where locally elevated depth of coverage suggests that assembly software may have compressed multiple 16S gene copies into a single locus.

### 16S rRNA diversity

**Figure 2:**
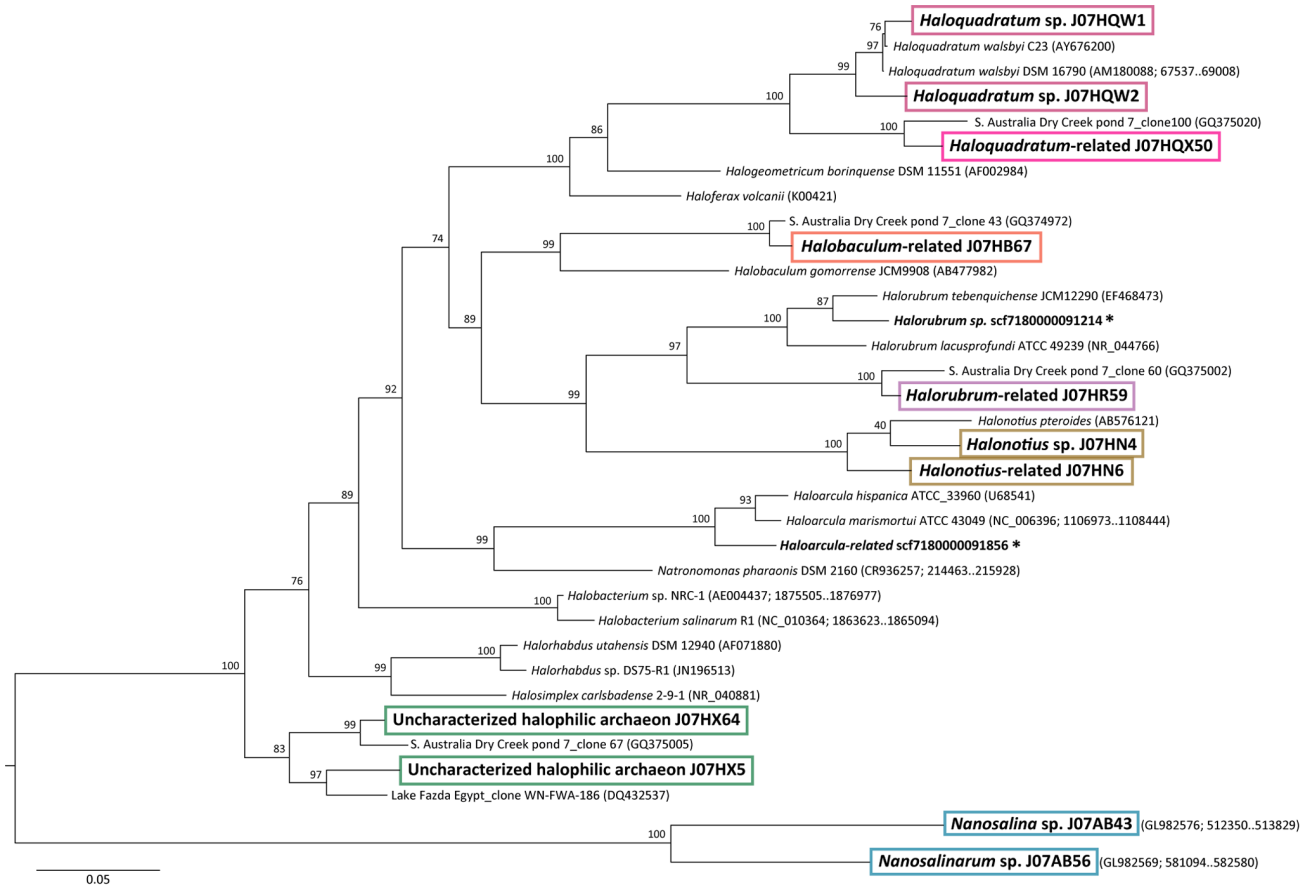
Phylogenetic distribution of archaeal 16S rRNA gene sequences in assembled scaffolds and population genomes. Names in bold indicate new 16S rRNA sequences identified in this study. Boxed names indicate sequences contained within Lake Tyrrell-specific population genomes. Asterisks indicate isolated individual sequences found on small scaffolds that were not associated with any assembled population genome.

Assembled sequences contained 34 distinct 16S rRNA gene sequences of 450 nt or longer, including 27 longer than 700 nt (**Table S4**). One scaffold contained a full-length 16S rRNA sequence that was 97% identical to cultured isolates of the halophilic bacterium *Salinibacter ruber*. The remaining 16S rRNA sequences were all archaeal, based on both BLAST searches against the Greengenes database and phylogenetic placement relative to characterized 16S rRNA gene sequences in a maximum-likelihood phylogenetic tree (**Figure 2**). Assembled archaeal 16S rRNA genes were distributed among seven broad phylogenetic groups, including class Nanohaloarchaea and relatives of previously sequenced isolates from Halobacterial genera *Haloquadratum*, *Halonotius*, *Halorubrum*, *Halobaculum*, *Halorhabdus*, and *Haloarcula*. Nearly all assembled 16S rRNA gene sequences had closer matches among uncharacterized environmental clones than sequenced isolate genomes.

A composite phylogenetic tree comparing archaeal 16S rRNA sequences from assembled scaffolds with the shorter, unassembled fragments (>350 nt) present in raw reads, placed >99% (1187/1202) of the unassembled read sequences into branches that were either basal, adjacent or identical to sequences represented by assembled scaffolds (**Figure S2a**). Assignment of basal positions to some of the shorter sequences present in unassembled reads reflects the unavailability of sufficient information to accurately resolve the placement of these 16S gene fragments. Several low-abundance clusters found in raw reads were not detected among the assembled scaffolds. These sequences were placed on branches adjacent to *Halovivax ruber*, *Haladaptatus paucihalophilus* and *Halobacterium salinarum*.

A similar, but less complete pattern of extended archaeal microdiversity was observed in archaeal PCR products when compared with assembled scaffold sequences (**Figure S2b**). A number of lineages present in both assembled scaffolds and raw metagenomic reads were missing from the PCR-generated 16S rRNA sequences. This result is consistent with previously described cases of universal archaeal primer bias preventing detection of novel archaeal taxa via PCR amplification [13], [37].

Eighty-five percent of the sequences amplified with archaeal primers matched assembled metagenomic scaffold sequences at 97% or greater sequence identity, suggesting membership in the same species. An additional 5% of the archaeal amplicons matched assembled sequences at 95–97% identity, most likely representing different species of the same genus. Eighty eight percent of the 16S rRNA amplicons obtained using bacterial primers matched cultured isolates of *Salinibacter ruber* at 97% or higher identity, confirming the dominance of this lineage among the bacterial community that was also observed in the assembled scaffolds.

### Scaffold Binning and Targeted Genome Reconstruction

**Figure 3:**
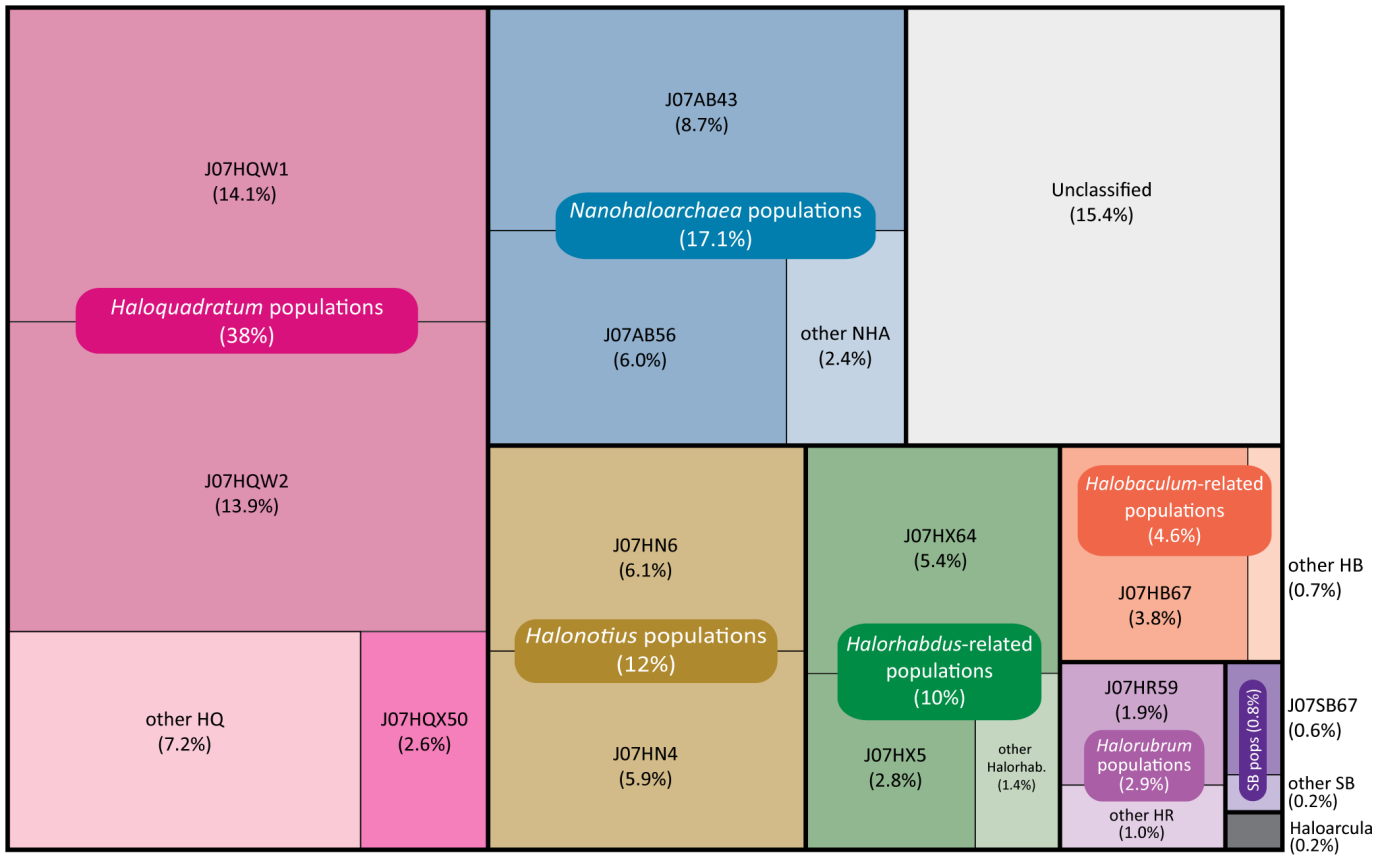
Relative abundance of microbial population groups. Colors correspond to taxonmically related microbial populations, including both assembled genome sequences and non-genomic scaffolds containing less abundant variant sequences. Percentage calculations include total number of assembled nucleotides in reads associated with each group, normalized for the group's average genome size. Percentage of unclassified sequences was calculated using an estimated genome size of 3 MB, the approximate abundance-weighted average for all other groups. Known viral and plasmid sequences, representing approximately 0.2% of assembled nucleotides, have been excluded from these calculations.

Eleven distinctive scaffold clusters were identified by applying the technique of Non-Metric Multidimensional Scaling to scaffold properties used for phylogenetic binning (**Figure S3**, **Table S5**). Each cluster was subjected to targeted iterative assembly yielding twelve genomes, eleven archaeal and one bacterial (**Table 1**). Each of these genomes represents the composite sampling of multiple individuals belonging to a genomically-similar population of closely related cells (species), approximating the dominant genotype extracted from a larger, polymorphic pool of closely related variants (strains). The treemap illustration presented in **Figure 3** shows the relative abundances of these populations in the context of all assembled scaffolds, organized according to taxonomically related population groups. This figure highlights the fact that each major population group contained multiple scaffold groups that could be identified as closely related to each other, but not necessarily assigned to specific genomes.

### Taxonomic Groups in Assembled Scaffolds

#### *Haloquadratum*-related populations J07HQW1, J07HQW2, and J07HQX50

Microbial populations related to cultured isolates of *Haloquadratum walsbyi* comprised 38% of the assembled Lake Tyrrell community sequences. Three distinct population genomes were reconstructed, named J07HQW1, J07HQW2, and J07HQX50. Based on16S rRNA sequence identity, J07HQW1 (99%) was more closely related to *H. walsbyi* cultured isolates than J07HQW2 (97%) or J07HQX50 (93%). These relationships were confirmed by adjacency in a maximum-likelihood phylogenetic tree (Figure 2). Mean assembly depths of coverage for both J07HQW1 and J07HQW2 (8.8-fold) were more than three-fold higher than for J07HQX50 (2.5-fold), suggesting considerably greater environmental abundance (Figure S4).

**Figure 4:**
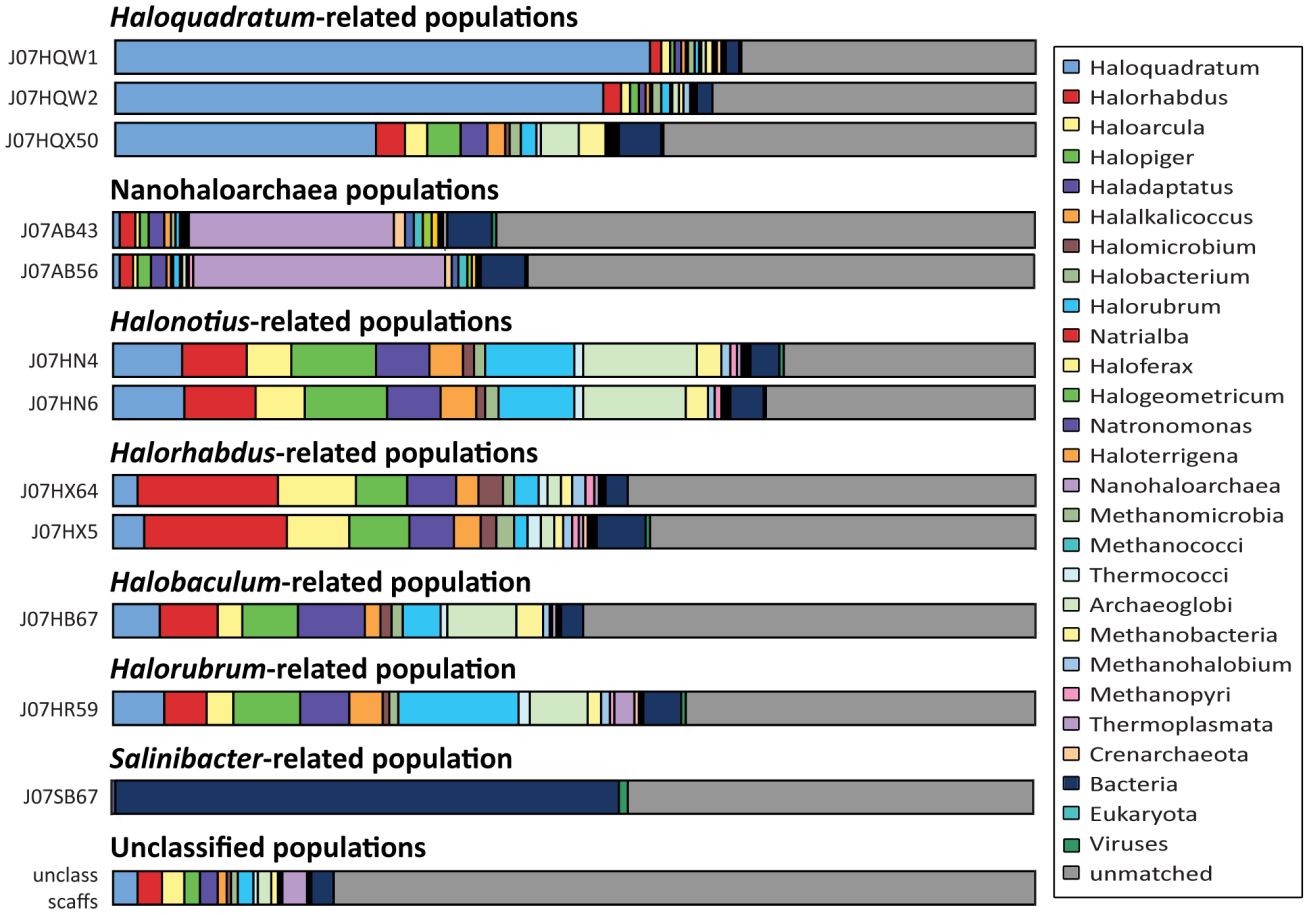
Phylogenetic distribution of protein BLAST matches for assembled population genomes and unclassified scaffolds. Taxonomic distribution of non-self matches versus the Genbank nr database were calculated using the DarkHorse algorithm at a filter threshold setting of 0.05, including only alignments covering at least 70% of both query and target sequences with an e-value of 1e-5 or better.

Authenticity of assembled 16S rRNA gene sequences from groups J07HQW1, J07HQW2, and J07HQX50 were corroborated by the presence of identical sequences in independent PCR clone libraries, as well as near-exact matches (>99% identity) in 16S rRNA sequences amplified by other investigators studying a different Australian hypersaline habitat [38]. In that study, sequences most closely matching J07HQX50 (phylogroup 2) were suggested to represent a separate genus from *H. walsbyi* strains C23 and DSM 16790. BLASTP analysis of predicted proteins in all three *Haloquadratum*-related genomes against Genbank nr reinforced taxonomic the relationships observed with 16S rRNA genes (**Figure 4**). The J07HQW1, J07HQW2, and J07HQX50 genomes all included a significant number of core gene matches to *H. walsbyi* cultured isolates. However, the overall percentage of predicted proteins with best matches to previously sequenced *Haloquadratum* genomes was less than half in J07HQX50 (28%) compared to J07HQW1 (58%), consistent with evolutionary diversification as a separate genus.

For populations like J07HQX50, where no physical data is available, distribution of scaffold reads between libraries obtained from 0.1 versus 0.8 µm filters can be used to obtain a rough estimate of cell size. Although it is not possible to determine exact cell size from read library distributions,high and low ends of the microbial size range sampled in Lake Tyrrell can be bracketed based on microscopically observed diameters of approximately 2 µm for the square cells of cultured *Haloquadratum* isolates (80% on 0.8 µm filters) and 0.6 µm for coccus-shaped environmental Nanohaloarchaea (<10% on 0.8 µm filters) [13].

Eighty percent of reads from scaffolds in all three *Haloquadratum*-related genomes were isolated from 0.8 µm pore filters, making them the largest cells in the current study. Finding 20% of the reads on 0.1 µm pore filters was initially unanticipated, based on the diameter of cultured *Haloquadratum* isolates and their known propensity to form multicellular aggregates. However, cultured *Haloquadratum* cells contain especially fragile internal gas vesicles, susceptible to collapse under pressures experienced during cellular concentration by filtration [39], [40]. In addition, nominal pore sizes reported for fiber-based filters are average values for a non-uniform size distribution that covers a wider range, explaining why some cells, especially those with flexible and/or asymmetric shapes, can routinely pass through filters with smaller than expected pore sizes.

#### Nanohaloarchaea populations J07AB43 and J07AB56

Sequences from archaeal class Nanohaloarchaea accounted for approximately 17% of the assembled microbial community, forming the second most abundant microbial group. Taxonomic binning of scaffolds from this group was facilitated by their significant divergence from other microbial groups in nucleotide G+C compositions, 16S rRNA gene sequences, predicted amino acid frequencies, and filter size distribution of reads [13]. Finding greater than 90% of the J07AB43 and J07AB56 reads in 0.1 µm pore filters agrees with previously reported cell diameters of approximately 0.6 microns, and suggests that they are the smallest cells whose genomes were assembled from the Lake Tyrrell environmental sequences.

#### *Halonotius*-related populations J07HN4 and J07HN6

The next most abundant population group, comprising approximately 12% of the community, contained two population genomes, J07HN4 and J07HN6. 16S rRNA gene sequences from these populations were 95–97% identical to *Halonotius pterides*, a cultured isolate for which no genome sequence is currently available [38], [41]. Despite differences in nucleotide composition between the two Lake Tyrrell *Halonotius*-like populations (63% versus 61% G+C), both shared similar amino acid composition profiles and taxonomic distributions of database matches for predicted proteins (Figure 4).

Based on scaffold read library distribution between 0.1 and 0.8 µm size fractions, *Halonotius*-like populations have the next smallest cells after Nanohaloarchaea in the Lake Tyrrell community. The percentage of 0.1 µm filter reads in J07HN6 (80%) was much higher than J07HN4 (50%) suggesting smaller cellular diameter in J07HN6. Although *Halonotius* cells have not been observed to undergo significant aggregation in culture, no data is currently available on whether this behavior might occur under natural conditions. Neither of the *Halonotius*-related genomes contain gas vesicle protein (gvp) synthesis genes, but both contain flagellar synthesis genes. Small flagellated cells and the absence of gas vesicles are consistent with light and electron micrograph observations of *H. pteroides* isolates in culture, which have cell diameters ranging between 0.7–1.5 µm and variable morphophologies including cocci, elongated rods and airfoil-like shapes [41].

#### *Halorhabdus*-related populations J07HX64 and J07HX5

Approximately 10% of assembled scaffold sequences formed a group most closely related to the genus *Halorhabdus*. The J07HX5 and J07HX64 genomes differed by 4% G+C, with 16S rRNA genes that were 96% identical to each other. J07HX64 matched an environmental 16S rRNA gene cloned from an Australian salt crystallizer (GQ375005) at 98% identity [38]. The closest environmental match to J07HX5 was to a 16S rRNA gene cloned from an Egyptian hypersaline lake (DQ432537), at 96% identity [42].

Predicted proteins from J07HX5 and J07HX64 shared similar amino acid composition signatures (**Table S5**) and similar taxonomic patterns of reference database BLASTP matches (**Figure 4**). *Halorhabdus* was the single most frequently matched genus at 15%, although several other Haloarchaeal genera matched at frequencies of 5–8%. Percentages of 0.1 µm pore filter reads comprising the J07HX5 (21%) and J07HX64 (24%) genome scaffolds suggest an effective cell size similar to *Haloquadratum*.

#### *Halobaculum*-related population J07HB67

Approximately 5% of the assembled Lake Tyrrell sequences were associated with a scaffold group named J07HB67. These scaffolds contain a 16S rRNA gene matching the genome of cultured isolate *Halobaculum gomorrense* at 92% identity. The J07HB67 16S rRNA gene is 99% identical to Australian salt crystallizer environmental clone GQ374998 (phylogroup 7) [38]. Approximately 33% of reads associated with J07HB67 populations were isolated from 0.1 µm pore filters, suggesting that cells from this population are larger than those of the *Halonotius* group, but smaller than *Haloquadratum*, J07HX5 and J07HX64. This finding is consistent with microscopic observations of *H. gomorrense*, whose rod-shaped cells measure 0.5–1 µm wide by 5–10 µm long [43].

#### *Halorubrum*-related populations

Assembled scaffolds from at least two *Halorubrum*-related populations, representing approximately 3% of the Lake Tyrrell microbial community, were linked by a common pattern of filter size distribution, percent G+C, amino acid sequence composition, and taxonomic classification of BLASTP hits against GenBank nr, in which *Halorubrum* was the most frequently matched genus. Two different *Halorubrum*-related 16S rRNA sequences were observed in assembled scaffolds, 90% identical to each other. Only one of these scaffold groups (J07HR59), representing approximately 2% of the assembled microbial community, was sufficiently abundant for population genome assembly. The J07HR59 16S rRNA sequence matched an environmental clone (GQ374972) described as Halorubrum-related phylogroup 4 at 97.4% identity [38], but J07HR59 and GQ374972 form a separate, independent branch from previously cultured isolate *Halorubrum* genomes (Figure 2). The other *Halorubrum*-related Lake Tyrrell population, representing approximately 1.0% of the assembled community, claded with previously cultured isolates, matching the *Halorubrum tebenquichense* 16S rRNA gene at 96% identity.

#### *Haloarcula* and other low abundance archaeal populations

Several small scaffolds containing solely archaeal 16S rRNA gene sequences were identified from populations with minimal genomic sampling (Table S4). These included two 16S rRNA sequences similar to cultured isolates of genus *Haloarcula*, at 3–4X depth of coverage. However, other scaffolds identifiable as *Haloarcula*-related were assembled at a coverage of 1.2 fold or less. *Haloarcula*-related 16S rRNA genes may have been more completely assembled than other loci from the same population due to multiple co-assembling gene copies; sequenced *Haloarcula* isolate genomes typically contain three 16S rRNA copies. Based on an estimated genome size of 3.9 Mbp, *Haloarcula*-related populations comprised approximately 0.2% of the assembled community, consistent with the lower depth of coverage of non-16S rRNA containing scaffolds.

#### *Salinibacter* population J07SB67

The only bacterial 16S rRNA sequence obtained from Lake Tyrrell metagenomic assembly matched cultured isolates of *Salinibacter ruber* at 98% identity, consistent with the observation that 3,480/3,958 (88%) of 16S rRNA sequences independently amplified using universal bacterial PCR primers matched cultured *Salinibacter* at 97% or higher identity. The assembled *Salinibacter* 16S rRNA gene was located on a small, 2,795 nucleotide scaffold, adjacent to a single predicted hypothetical protein. However, more than 400 additional scaffolds, ranging in size from 1,000–19,000 nucleotides, shared patterns of BLAST match taxonomy, nucleotide composition, and predicted amino acid composition consistent with assignment to a *Salinibacter*-related species.

Targeted assembly of the *Salinibacter*-related scaffold group yielded an incomplete genome of only 1.2 Mbp, versus 3.6 MB for previously sequenced *Salinibacter* isolates (33.3% genome coverage) [44]. Thirty-nine percent of highly conserved bacterial core proteins present in both cultured *Salinibacter* isolate genomes were recovered, consistent with total genome length. Depth of coverage for *Salinibacter*-related scaffolds averaged 1.5 fold, corresponding to a nucleotide abundance of approximately 0.6 percent of the microbial community.

#### Viral and “Plasmidome” community sampling

Despite the use of sample preparation methods designed to capture only cells between 0.1 and 3 µm in diameter, a group of 142 small scaffolds, representing approximately 0.2% of assembled nucleotides, contained DNA fragments that appear viral in origin. These fragments ranged in size from 1,000 to 25,000 nucleotides in length, with compositions varying between 35–71% G+C. Most of these putative viral scaffolds were reconstructed exclusively from 0.1 µm filter reads. These results are consistent with non-specific retention of viral particles on filter surfaces and/or recovery of phage genomes from infected cells during sample preparation. Predicted proteins in these scaffolds included BLAST matches to viral groups previously shown to be abundant in hypersaline waters, including BJ1-like Siphoviridae and PhiCh-like Myoviridae [45], [46], [47], [48], [49], [50]. Recovered data were insufficient to determine whether or not these sequences were integrated as prophage in microbial genomes.

Forty scaffolds ranging in size from 1–50 kbp, comprising approximately 0.2% of assembled nucleotides, contained genes encoding p4 plasmid primase, suggesting that they may be archaeal plasmid sequences. Two additional scaffolds contained matches to the *Salinibacter ruber* plasmid protein init Rep_3. Nucleotide composition of putative plasmid scaffolds ranged from 49–66% G+C, at 1.1–12.8 fold depth of coverage, suggesting association with both dominant and rare community members. However, most putative plasmid scaffolds could not be confidently assigned to a specific host organism, and contained few predicted proteins similar to previously sequenced database representatives. Plasmid numbers in cultured halophilic Archaea and Bacteria vary between zero (e.g *Haloquadratum walsbyi* DSM 16790) and seven (e.g. *Haloarcula marismortui* ATCC 43049), with sizes ranging from <2 Kbp (*Halobacterium salinarum*, NC_002121) to >600 Kbp (*Haloferax volcanii* DS2, NC_013966). This extremely wide variability makes it difficult to determine the extent to which the plasmid scaffolds we observed represent partial versus complete sequences.

#### Unclassified Sequences

Approximately 15% of assembled scaffold sequences could not be confidently assigned to any of the groups described above. The low assembly coverage and short sequence lengths in these scaffolds most likely encompass not only less abundant members of the community, but also partial, incomplete fragments corresponding to polymorphic insertions, deletions, mutations, and rearrangements between related strains. Seventy-six percent of predicted protein sequences in the unclassified scaffold group failed to match any sequences in Genbank nr. Database matches were predominately archaeal in origin, including the same reference organisms as assembled consensus population genomes (Figure 4).

To estimate the extent to which unclassified scaffolds might represent uncaptured functional diversity within the community, all predicted proteins from the original composite assembly, including both classified and unclassified sequences, were screened for matches to PFAM, COG, and KEGG protein database patterns. At least one pattern was found in 31,696 of 62,918 predicted proteins. Even though unclassified scaffolds comprise 15% of total assembled nucleotides, they contained only 326 patterns absent from the classified data set, corresponding to 7.5% of the overall pool. Classified scaffolds contained 92.5% of all protein patterns detected (5,197 proteins). Protein domain patterns unique to the unclassified scaffolds included a large number of viral-related functional elements, as well as low complexity short repeats characteristic of incomplete protein fragments, suggesting that this group contains an over-representation of partial genes and viral fragments.

To eliminate potential bias due to the highly conserved nature of COG, KEGG, and PFAM patterns, unsupervised Markov algorithm clustering was also performed on all 62,918 predicted proteins in the initial combined assembly. Based on frequencies of these unsupervised clusters, Chao and Ace estimators indicate that assembled scaffolds include greater than >90% of the expected functional diversity in the sampled community. Classified scaffolds contained 4,432 of the 5,242 clusters observed, with only 810 clusters occurring uniquely in the unlassified scaffold set. Close agreement between the percentage of protein clusters (84.6%) and total nucleotides incorporated in assembled scaffolds (84.5%) supports use of the classified data set as a representative sample of functional diversity within the community.

### Population Distribution of Community Functions

**Figure 5:**
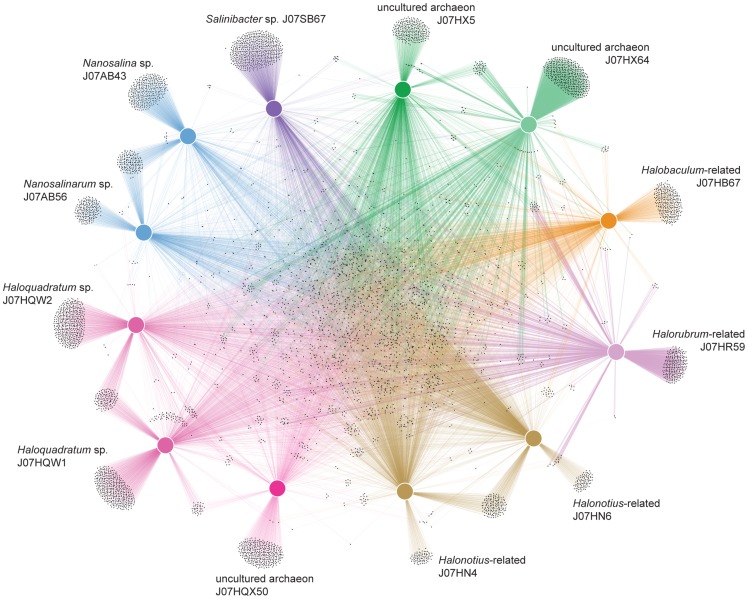
Metabolic connectivity graph showing community distribution of protein family clusters. Cohesive populations are shown as similarly colored nodes and vectors according to numbers of shared features, based on unsupervised protein family clustering of 12 habitat-specific genomes.

Markov algorithm clustering was applied to all 31,062 predicted proteins from the twelve Lake Tyrrell genomes, generating 6,591 protein families. Protein family clusters shared between different populations were plotted as connections in a network representation (**Figure 5**). Highly interconnected clusters, converging at the center of the diagram, include both universal housekeeping genes and habitat-specific adaptive capabilities. Functions broadly shared among all taxonomic groups suggest a common aerobic, heterotrophic lifestyle. Protein families conserved in all 11 archaeal populations also include UV damage repair endonucleases, peroxiredoxins and thioredoxins, halocyanins, Ca^2+^/Na^+^ antiporters, and type IS605 OrfB family transposases.

Population-specific protein families located at the periphery of **Figure 5** capture functional novelty of both individual genomes and closely related taxonomic groups relative to the rest of the community. **Table 2** compares numbers of unique clusters found in each of the twelve consensus genomes. The population with the greatest number of unshared protein families is *Salinibacter*, the only bacterium in the group, even though the assembled genome was less than 40% complete. The two Nanohaloarchaeal genomes J07AB43 and J07AB56 also contained many unique clusters, both individually and shared between them.

**Table 2:** Population-unique protein family clusters.

Genome name	num unique clusters	total num genome clusters	pct. unique clusters
*Salinibacter* sp. J07SB67	581	1,639	35%
*Nanosalina* sp. J07AB43	366	1,678	22%
uncultured archaeon sp. J07HX64	441	3,047	14%
*Nanosalinarum* sp. J07AB56	184	1,410	13%
*Halorubrum* sp. J07HR59	232	1,839	13%
*Haloquadratum* sp. J07HQX50	351	2,872	12%
uncultured archaea sp. J07HX5	258	2,139	12%
*Haloquadratum* walsbyi str. J07HQW1	403	3,584	11%
*Haloquadratum* walsbyi str. J07HQW2	433	3,855	11%
*Halobaculum* sp. J07HB67	296	2,846	10%
*Halonotius* sp. J07HN6	90	2,913	3%
*Halonotius* sp. J07HN4	81	3,229	3%
**total**	**3,716**	**31,051**	**12%**

Although each of the three *Haloquadratum* genomes had more than 350 unique clusters, these numbers were similar to other Lake Tyrrell Haloarchaeal populations when normalized for genome size. Numbers of novel clusters found in *Haloquadratum*-related populations suggest more diverse protein functions than other community members, but most likely also include a higher number of pseudogenes, as observed previously in *Haloquadratum* isolate genomes [51], [52]. In contrast to *Haloquadratum*, *Halonotius* populations J07HN4 and J07HN6 contain surprisingly few novel protein clusters in each individual genome, despite 16S rRNA sequences that are more divergent from each other than the J07HQW1 and J07HQW2 genomes. Unique functional properties of the *Halonotius* group are captured instead in clusters shared between J07HN4 and J07HN6.

Many protein families shared between different community members contain only sequences whose function cannot be predicted from bioinformatic inference. Proteins of unknown function are more abundant among population-specific protein families than in more widely distributed clusters. However, even confidently annotated proteins sometimes generate multiple clusters with similar descriptions, and may correspond to protein isoform variants with unknown but possibly significant differences in biological activity.

## Discussion

In this study we have captured the taxonomic diversity, population abundance, and functional properties associated with both broad phylogenetic groups and individual microbial populations in a mixed, natural ecosystem community. Reconstruction of 12 habitat-specific population genomes from a single pool of metagenomic sequencing reads demonstrates the value of combining *de novo* assembly with iterative, multi-dimensional phylogenetic binning. This approach proved particularly useful in characterizing previously undescribed novel organisms, avoiding problematic issues of amplification primer bias and variable 16S rRNA gene copy number in divergent populations. Eight reconstructed genomes represented species with no previously cultured isolates, including populations comprising 2–14% of the sampled microbial community. Ten of the twelve genomes were nearly complete, in assemblies of seven or fewer scaffolds.

Each of these population genomes represents a composite sequence constructed from multiple, closely related individual cells, providing a set of core gene models and operon structures common to most members of the population. These genomes do not include peripheral pan-genomic content that is unique to individual strains. Regions of significant population divergence (intra-species heterogeneity) are incorporated as gaps in larger scaffolds and/or separate shorter overlapping scaffolds with lower read coverage. The composite sequences we have obtained by community metagenomic assembly cannot be expected to furnish the same level of detail and accuracy as the closed, finished genome of an individual isolate, yet their ability to deliver full length genes in cellular context has provided important new insights into community structure, novel taxa, and compartmentalized protein functional associations that could not be obtained from unassembled reads alone.

Although Sanger technology was the primary source of reads for this study, the subtractive taxonomic enrichment strategies we have developed could also be applied to metagenomic assemblies using paired-end reads obtained by more contemporary platforms such as Illumina. Our *de novo* assembly procedures were especially effective in faciliting genome recovery for populations (species) with no closely related sequenced relatives. Assembly quality was improved as data complexity was reduced and the accuracy of read binning enhanced by iterative, scaffold-based read selection using multiple, independent parameters. These parameters included uniform nucleotide composition, depth of coverage, taxonomic distribution of BLASTP database matches, and amino acid composition of predicted proteins. Read distribution frequencies from overlapping libraries obtained using different filter pore sizes provided an additional source of independent information to help distinguish difficult-to-separate groups and verify assembly fidelity, as well as offering a novel opportunity to estimate physical cell size of uncharacterized organisms relative to other members of the community.

Archaea greatly outnumbered Bacteria in the Lake Tyrrell hypersaline ecosystem, as previously reported for other extreme hypersaline environments [14], [53]. Although relatives of *Haloquadratum walsbyi* were the most abundant taxonomic group, comprising approximately 38% of the community, nearly 47% of the assembled sequences were derived from a combination of Nanohaloarchaea (17%) and relatives of the Haloarchaeal genera *Halonotius* (12%), *Halorhabdus* (10%), *Halobaculum* (4.6%), *Halorubrum* (2.9%), and *Haloarcula* (0.2%). Based on historical accounts of other hypersaline habitats [52], [54], [55], diversity within the *Haloquadratum*-related population was higher than expected, including at least three different species from two different genera.

The 62,918 environmental genes recovered from the assembled metagenomic sequences were estimated to encompass more than 90% of the functional diversity present in the community. The construction of multiple habitat-specific Lake Tyrrell population genomes has enabled genome-wide assignment of functional activities to specific individual organisms of known abundance in the community. These assignments provide new opportunities to begin comparing shared and novel protein families across related and divergent co-occurring populations adapted to the same environmental conditions with a level of organism-specific context that would not be possible with unassembled reads alone.

The relatively constrained metabolic repertoire of broadly shared protein functional families in the Lake Tyrrell community may be linked to physicochemical uniformity in the shallow, aquatic hypersaline environment from which organisms were sampled. The common evolutionary history of halophilic Archaea adapted to extreme salinity may also play a role. It has been speculated that abundances of different microbial populations under these conditions might be driven more by top down forcing dynamics, for example protozoan predation and/or viral infections, rather than nutrient availability [56]. The current study does not include seasonal fluctuations in temperature, salinity and nutrient inputs, which might reveal greater diversity over longer time scales. The availability of new habitat-specific reference genomes from the Lake Tyrrell ecosystem provides new reference data to track these populations over time and space at the level of both genes and genomes.

Functional genes and metabolic processes unique to individual populations may also provide information useful in designing cultivation methods for previously uncultured organisms, including the possibility of mixed co-cultures to accommodate natural symbiotic or co-dependent trophic relationships. The potential utility of this approach is illustrated by the observation that strains of *Haloquadratum walsbyi*, notoriously difficult to grow in isolate culture, form significantly larger colonies in the presence of *Salinibacter ruber* [57]. Although *Salinibacter*-related populations comprise only a small percentage of the ecosystem described here, *Haloquadratum* abundance could be driven by similar nutritional complementation provided by alternative members of the community.

The new genomes described in this study expand opportunities to identify novel phylogroups in other environments, providing new templates for fragment recruitment and assembly, as well as group-specific probes for *in situ* quantitation. Organisms previously identified by 16S rRNA gene sequences alone can now be prioritized as targets for more detailed investigations based on functional, as well as taxonomic information. Furthermore, the assembly of habitat-specific genomes provides an important foundation to decipher genotype-phenotype relationships based on metatranscriptomic and metaproteomic investigations in similar environments. The simultaneous interrogation and synthesis of composite data from multiple microbial populations in natural ecosystems will provide the comprehensive level of genotypic and phenotypic data necessary to model synergistic activities of community members, while contributing to an enhanced understanding of the ecology and evolution of environmental microbial species.

## Supporting Information

Table S1 Water chemistry of Lake Tyrrell sampling site. Located at 35°19′12.24S 142°48′00.45E.
(PDF)

Table S2 Summary of metagenomic sequencing libraries used in this study. Average read length is shown ± standard deviation.
(PDF)

Table S3 Assembly statistics for combined Sanger metagenomic libraries using Celera Assembler version 5.4. Assembly parameters used were as follows: utgErrorRate = 0.10; ovlErrorRate = 0.10; cnsErrorRate = 0.10; cgwErrorRate = 0.12; utgBubblePopping = 0; utgGenomeSize = 500000; merSize = 15; doFragmentCorrection = 0; doExtendClearRanges = 1; doResolveSurrogates = 1; Unitigger parameter –j = −20.
(PDF)

Table S4 Assembled 16S rRNA sequences and their closest database matches to environmental clones and cultured isolates. Matches were required to have BLAST alignments to previously identified 16S rRNA genes of 450 nt or longer, with e-value <1e-7 and 80% or greater sequence identity between query and subject. Part A shows 16S rRNA gene sequences obtained in targeted genomic assemblies. Part B shows additional 16S rRNA gene sequences observed in scaffolds obtained by composite assembly of all Sanger reads.
(PDF)

Table S5 Distinctive properties of major scaffold clusters. Percentages are based on taxonomic classifications of all predicted protein tophit matches to Genbank nr, as determined using the DarkHorse algorithm at a filter threshold setting of 0.05, including only alignments covering at least 70% of both query and target sequences with an e-value of 1e-5 or better.
(PDF)

Table S6 Estimated genome completeness. Based on presence/absence of 53 conserved genes in assembled archaeal composite population genomes.
(PDF)

Figure S1 **Bioinformatic Analysis Pipeline.**
(PDF)

Figure S2 **Phylogenetic trees showing abundance of clustered archaeal 16S rRNA sequences from (A) unassembled reads and (B) PCR-amplified clone libraries.** A maximum likelihood archaeal reference tree was constructed using FastTree [1], based on full-length 16S genes from isolate genomes and environmental clones from Genbank nt, supplemented with sequences obtained from Lake Tyrrell assembled scaffolds (highlighted in yellow). Additional partial 16S rRNA sequences from Lake Tyrrell were inserted into the reference tree using pplacer version v1.1 (model GTR, fig-eval-all) [2] and visualized using Archaeopteryx 0.968 [3]. Part A shows placement of un-amplified raw metagenomic reads containing 16S gene sequences. Part B shows placement of PCR-amplified 16S rRNA clones. Numbers at nodes indicate confidence values estimated by FastTree for the reference tree. Red lines indicate branches where Lake Tyrrell sequences were observed. The thickness of each red line is proportional to the number of Lake Tyrrell sequences associated with that branch, ranging from one in the thinnest line to 74 in the thickest line.
(PDF)

Figure S3 **Non-metric multidimensional scaling plot illustrating distinctive scaffold groups.** Scaffolds >5 Kb from the composite Sanger assembly were subjected to non-metric multidimensional scaling analysis using Primer 1.6, with Euclidean distance, 25 random starts, Krustal fit scheme 1, and minimum stress value 0.01 for the 13 parameters shown in Table S5. Axes shown are arbitrary units of composite clustering, although the X axis appears to be dominated by nucleotide percent G+C. Scaffolds associated with major taxonomic groups are highlighted with colored symbols. Small grey dots indicate scaffolds that could not be unambiguously classified into major groups.
(PDF)

Figure S4 **Rank abundance of assembled microbial populations based on depth of coverage.**
(PDF)
