# Supplementary material for: Assembly-Driven Community Genomics of a Hypersaline Microbial Ecosystem
Source: PLoS One. 2013 Apr 18;8(4):e61692. doi: 10.1371/journal.pone.0061692 (PMC3630111; doi:10.1371/journal.pone.0061692)
Supplement: Table S1 — Water chemistry of Lake Tyrrell sampling site. Located at 35°19′12.24S 142°48′00.45E. (PDF) [file pone.0061692.s001.pdf]

Supporting Table S1. Water chemistry of Lake Tyrrell sampling site, located at 35°19' 12.24S 142°48' 00.45E.

collection date	water temp (°C)	Salinity (ppt)	TDS (wt%)	pH
1/23/07	22	127	31	7.23
1/25/07	28	132	31	7.09
