# Supplementary material for: Assembly-Driven Community Genomics of a Hypersaline Microbial Ecosystem
Source: PLoS One. 2013 Apr 18;8(4):e61692. doi: 10.1371/journal.pone.0061692 (PMC3630111; doi:10.1371/journal.pone.0061692)
Supplement: Table S2 — Summary of metagenomic sequencing libraries used in this study. Average read length is shown ± standard deviation. (PDF) [file pone.0061692.s002.pdf]

Supporting Table S2. Summary of metagenomic sequencing libraries used in this study. Average read length is shown ± standard deviation.

**Library name**	**Retention Filter**	**Collection Date**	**Library type**	**Sequencing platform**	**Number of reads**	**Avg. read length**
ABM	0.8 µm	1/23/2007	8-10 kb plasmid	Sanger	81,197	776 ± 157
ABL	0.8 µm	1/23/2007	40 kb fosmid	Sanger	43,490	599 ± 230
CBM	0.8 µm	1/25/2007	8-10 kb plasmid	Sanger	81,032	781 ± 155
CBL	0.8 µm	1/25/2007	40 kb fosmid	Sanger	33,029	678 ± 191
AAM	0.1 µm	1/23/2007	8-10 kb plasmid	Sanger	95,988	699 ± 198
CAM	0.1 µm	1/25/2007	8-10 kb plasmid	Sanger	92,242	747 ± 179
**Sanger Total**					**426,978**	**727 ± 180**

**ABT**	0.8 µm	1/23/2007	-	454 Titanium	994,337	362 ± 96
**CAT**	0.1 µm	1/25/2007	-	454 Titanium	205,925	401 ± 119
**454 total**					**1,200,262**	**368 ± 100**
