# Supplementary material for: Assembly-Driven Community Genomics of a Hypersaline Microbial Ecosystem
Source: PLoS One. 2013 Apr 18;8(4):e61692. doi: 10.1371/journal.pone.0061692 (PMC3630111; doi:10.1371/journal.pone.0061692)
Supplement: Table S3 — Assembly statistics for combined Sanger metagenomic libraries using Celera Assembler version 5.4. Assembly parameters used were as follows: utgErrorRate = 0.10; ovlErrorRate = 0.10; cnsErrorRate = 0.10; cgwErrorRate = 0.12; utgBubblePopping = 0; utgGenomeSize = 500000; merSize = 15; doFragmentCorrection = 0; doExtendClearRanges = 1; doResolveSurrogates = 1; Unitigger parameter –j = −20. (PDF) [file pone.0061692.s003.pdf]

Supporting Table S3. Assembly statistics for combined Sanger metagenomic libraries using Celera Assembler version 5.4. Assembly parameters used were as follows: utgErrorRate=0.10; ovlErrorRate=0.10; cnsErrorRate=0.10; cgwErrorRate=0.12; utgBubblePopping=0; utgGenomeSize=500000; merSize=15; doFragmentCorrection=0; doExtendClearRanges=1; doResolveSurrogates=1; Unitigger parameter –j = –20.

total num nucleotides	286,258,541
num reads	426,864
num scaffolds	15,008
scaffold N50	29,346
max scaffold length	2,982,959
num predicted proteins	62,915
num placed reads	282,583
% bp placed	69.60%
% reads placed	66.20%
