# Supplementary material for: Assembly-Driven Community Genomics of a Hypersaline Microbial Ecosystem
Source: PLoS One. 2013 Apr 18;8(4):e61692. doi: 10.1371/journal.pone.0061692 (PMC3630111; doi:10.1371/journal.pone.0061692)
Supplement: Table S4 — Assembled 16S rRNA sequences and their closest database matches to environmental clones and cultured isolates. Matches were required to have BLAST alignments to previously identified 16S rRNA genes of 450 nt or longer, with e-value <1e-7 and 80% or greater sequence identity between query and subject. Part A shows 16S rRNA gene sequences obtained in targeted genomic assemblies. Part B shows additional 16S rRNA gene sequences observed in scaffolds obtained by composite assembly of all Sanger reads. (PDF) [file pone.0061692.s004.pdf]

Supporting Table S4. Assembled 16S rRNA sequences and their closest database matches to environmental clones and cultured isolates. Matches were required to have BLAST alignments to previously identified 16S rRNA genes of 450 nt or longer, with e-value < 1e-7 and 80% or greater sequence identity between query and subject. Part A shows 16S rRNA gene sequences obtained in targeted genomic assemblies. Part B shows additional 16S rRNA gene sequences observed in scaffolds obtained by composite assembly of all Sanger reads.

A

Genome	Scaffold id	16S sequence length	Closest Environmental match	Environ match pct identity	Closest cultured isolate match	Isolate match pct identity
**J07HQW1 copy 1**	scf56329	1472	GQ374993 - Baj_clone41 (Australia)	99.8	Haloquadratum walsbyi DSM 16790	99.1
**J07HQW1 copy 2**	scf56329	1472	GQ374996 - Baj_clone47 (Australia)	99.0	Haloquadratum walsbyi DSM 16790	98.6
**J07HQW2 copy 1**	scf02443	1473	GQ374961 - Cry7_clone4 (Australia)	99.1	Haloquadratum walsbyi DSM 16790	97.5
**J07HQW2 copy 2**	scf02443	709	GQ374961 - Cry7_clone4 (Australia)	99.3	Haloquadratum walsbyi DSM 16790	97.6
**J07HQX50**	scf01540	1473	GQ375020 - Cry7_clone100 (Australia)	96.9	Haloquadratum walsbyi DSM 16790	93.5
**J07AB43**	scf30744	1479	GQ375009 - Cry7_clone76 (Australia)	99.4	none	-
**J07AB56**	scf39101	1486	GQ375000 - Cry7_clone54 (Australia)	99.5	none	-
**J07HN4**	scf01348	1469	GQ374992 - Baj_clone38 (Australia)	96.1	Halonotius pteroides 302698938	96.1
**J07HN6**	scf01851	1468	FN994966 - SFF1C121 (Tunisia)	97.9	Halonotius pteroides 302698938	95.4
**J07HR59**	scf01382	1470	GQ375002 - Cry7_clone60 (Australia)	97.4	Halorubrum tebenquichense JCM12290	90.6
**J07HX5**	scf22092	1468	DQ432537 - WN-FWA-186 (Egypt)	96.0	Halosimplex carlsbadense	91.0
**J07HX64**	scf96483	1010	GQ375005 - Cry7_clone67	98.0	Halorhabdus utahensis AX-2	92.3

Genome	Scaffold id	16S sequence length	Closest Environmental match	Environ match pct identity	Closest cultured isolate match	Isolate match pct identity
			(Australia)			
**J07HB67**	scf98878	1360	GQ374972 - Cry7_clone43 (Australia)	99.0	Halobaculum gomorrense str JCM 9908	90.0
**J07SB67**	scf87461	1495	HQ157666 - TSHNAE18 (Tunisia)	98.2	Salinibacter ruber DSM 13855	97.3

B

Taxonomic group	Scaffold id	16S sequence length	Closest Environmental match	Environ match pct identity	Closest cultured isolate match	Isolate match pct identity
**Haloquadratum**	scf98861	1474	GQ374961 - Cry7_clone4 (Australia)	99.2	Haloquadratum walsbyi 640715938	98.0
**J07AB43**	scf98840	1448	GQ375009 - Cry7_clone76 (Australia)	99.7	none	-
**J07AB56**	scf98837	1383	GQ375000 - Cry7_clone54 (Australia)	99.9	none	-
**J07HN4**	scf98475	1352	GQ374992 - Baj_clone38 (Australia)	97.3	Halonotius pteroides 302698938	97.1
**Haloarcula**	scf91856	1295	EU722673 - SA21 (Spain)	97.3	Haloarcula hispanica str HLR2	96.6
**J07AB43**	scf86038	1039	GQ375009 - Cry7_clone76 (Australia)	95.6	none	-
**J07HR59**	scf91079	1038	GQ375002 - Cry7_clone60 (Australia)	98.1	Halorubrum tebenquichense JCM12290	92.2
**Halorubrum**	scf91214	839	GQ375031 - LDS1_clone58 (Australia)	98.5	Halorubrum tebenquichense	96.2
**J07HN4**	scf98871	796	GQ374959 - Baj_clone12 (Australia)	99.4	Halonotius pteroides 302698938	95.2
**J07HX5**	scf93563	793	GQ375002 - Cry7_clone60 (Australia)	94.4	Natronomonas pharaonis DSM 2160	90.6

Taxonomic group	Scaffold id	16S sequence length	Closest Environmental match	Environ match pct identity	Closest cultured isolate match	Isolate match pct identity
**J07HX5**	scf98852	780	GQ375002 - Cry7_clone60 (Australia)	94.0	Halomicrobium mukohataei DSM 12286	91.4
**HQX50**	scf98854	738	GQ375020 - Cry7_clone100 (Australia)	99.9	Haloquadratum walsbyi DSM 16790	92.0
**Haloquadratum**	scf98861	712	GQ374961 - Cry7_clone4 (Australia)	99.2	Haloquadratum walsbyi DSM 16790	97.5
**HQX50**	scf98854	653	GQ374945 - LDS1_clone37 (Australia)	99.7	Haloquadratum walsbyi DSM 16790	95.1
**Unclassified**	scf93739	650	GQ375041 - LDS1D3.3 (Australia)	100.0	Halorientalis regularis strain TBN19	96.0
**Haloarcula**	scf84445	631	JN714439 - XKL43 (China)	96.8	Haloarcula hispanica str HLR2	95.5
**J07HN6**	scf98809	574	FN994966 - SFF1C121 (Tunisia)	99.5	Halonotius pteroides 302698938	95.0
**Haloquadratum**	scf98859	560	GQ374990 - Baj_clone34 (Australia)	99.8	Haloquadratum walsbyi DSM 16790	99.1
**Haloquadratum**	scf98874	512	GQ374952 - Baj_clone1 (Australia)	99.6	Haloquadratum walsbyi DSM 16790	98.4
**J07HX64**	scf98875	480	GQ375005 - Cry7_clone67 (Australia)	98.1	Haloferax zhejiangensis ZJ206	92.1
