# Supplementary material for: Assembly-Driven Community Genomics of a Hypersaline Microbial Ecosystem
Source: PLoS One. 2013 Apr 18;8(4):e61692. doi: 10.1371/journal.pone.0061692 (PMC3630111; doi:10.1371/journal.pone.0061692)
Supplement: Table S5 — Distinctive properties of major scaffold clusters. Percentages are based on taxonomic classifications of all predicted protein tophit matches to Genbank nr, as determined using the DarkHorse algorithm at a filter threshold setting of 0.05, including only alignments covering at least 70% of both query and target sequences with an e-value of 1e-5 or better. (PDF) [file pone.0061692.s005.pdf]

Supporting Table S5. Distinctive properties of major scaffold clusters. Percentages are based on taxonomic classifications of all predicted protein tophit matches to Genbank nr, as determined using the DarkHorse algorithm at a filter threshold setting of 0.05, including only alignments covering at least 70% of both query and target sequences with an e-value of 1e-5 or better.

group	pct G+C	pct reads 0.1 filt	avg read depth	pct Halo-quadrataum	pct Nano-haloarchaea	pct Halo-rabdus	pct Halo-ferax	pct Halo-rubrum	pct Salini-bacter	pct LYS	pct THR	pct ALA	pct GLU
**Haloquadratum**	48	20%	8.6	79%	0%	2%	1%	1%	0%	2.3	7.5	9.0	7.7
**HQX50**	50	20%	2.5	44%	1%	5%	7%	2%	0%	1.4	4.3	5.6	4.8
**Halonotius HN4**	61	59%	3.8	10%	1%	12%	15%	12%	0%	1.7	6.5	10.1	7.2
**Halonotius HN6**	63	81%	4.6	9%	0%	12%	17%	12%	0%	1.7	6.2	9.9	7.2
**Nanosalina J07AB43**	43	94%	6.4	1%	60%	3%	1%	2%	0%	6.0	5.0	5.8	10.5
**Nanosalinarum J07AB56**	56	91%	4.7	1%	53%	4%	0%	1%	0%	4.0	5.3	7.1	10.2
**HX5**	60	21%	2.9	5%	1%	28%	2%	3%	0%	1.4	6.3	9.8	6.1
**HX64**	64	24%	3.6	5%	1%	27%	3%	4%	0%	1.3	6.6	9.4	7.9
**HB67**	67	33%	2.9	10%	1%	12%	14%	8%	0%	1.3	7.5	11.4	7.7
**Halorubrum**	60	25%	2.4	7%	0%	7%	14%	32%	0%	0.9	3.1	4.9	3.7
**Salinibacter**	66	44%	2.2	0%	0%	0%	0%	0%	92%	0.8	2.7	4.4	3.0
