# Supplementary material for: Assembly-Driven Community Genomics of a Hypersaline Microbial Ecosystem
Source: PLoS One. 2013 Apr 18;8(4):e61692. doi: 10.1371/journal.pone.0061692 (PMC3630111; doi:10.1371/journal.pone.0061692)
Supplement: Table S6 — Estimated genome completeness. Based on presence/absence of 53 conserved genes in assembled archaeal composite population genomes. (PDF) [file pone.0061692.s006.pdf]

Supporting Table S6. Estimated genome completeness based on presence/absence of 53 conserved genes in assembled archaeal composite population genomes.

Function id	Function name	J07HQW1	J07HQW2	J07HQX50	J07HN4	J07HN6	J07HR59	J07HX64	J07HX5	J07HB67
total		53	52	48	53	53	44	49	28	50
pct complete		100%	98%	91%	100%	100%	83%	92%	53%	94%
COG0008	Glutamyl- and glutaminyl-tRNA synthetases	+	+	+	+	+	+	+	+	+
COG0012	Predicted GTPase, probable translation factor	+	+	+	+	+	+	+	+	+
COG0013	Alanyl-tRNA synthetase	+	+	+	+	+	+	+	+	+
COG0016	Phenylalanyl-tRNA synthetase alpha subunit	+	+	+	+	+	+	+	+	+
COG0018	Arginyl-tRNA synthetase	+	+	+	+	+	+	+	+	+
COG0024	Methionine aminopeptidase	+	+	+	+	+	+	+	+	+
COG0048	Ribosomal protein S12	+	+	+	+	+	+	+	-	+
COG0049	Ribosomal protein S7	+	+	+	+	+	+	+	-	+
COG0051	Ribosomal protein S10	+	+	+	+	+	+	+	+	+
COG0052	Ribosomal protein S2	+	+	+	+	+	-	+	+	+
COG0060	Isoleucyl-tRNA synthetase	+	+	+	+	+	-	+	+	+
COG0071	Molecular chaperone (small heat shock protein)	+	+	+	+	+	+	+	+	+
COG0080	Ribosomal protein L11	+	+	+	+	+	-	+	+	+
COG0081	Ribosomal protein L1	+	+	+	+	+	+	+	+	+
COG0085	DNA-directed RNA polymerase, beta subunit/140 kD subunit	+	+	+	+	+	+	+	-	+
COG0086	DNA-directed RNA	+	+	+	+	+	+	+	-	+

Function id	Function name	J07HQW1	J07HQW2	J07HQX50	J07HN4	J07HN6	J07HR59	J07HX64	J07HX5	J07HB67
	polymerase, beta' subunit/160 kD subunit									
COG0087	Ribosomal protein L3	+	+	+	+	+	-	+	-	+
COG0088	Ribosomal protein L4	+	+	+	+	+	+	+	-	+
COG0090	Ribosomal protein L2	+	+	+	+	+	+	+	-	+
COG0091	Ribosomal protein L22	+	+	+	+	+	+	+	-	+
COG0092	Ribosomal protein S3	+	+	+	+	+	+	+	-	+
COG0093	Ribosomal protein L14	+	+	+	+	+	+	+	-	+
COG0094	Ribosomal protein L5	+	+	-	+	+	-	-	-	+
COG0096	Ribosomal protein S8	+	+	+	+	+	-	+	-	+
COG0097	Ribosomal protein L6P/L9E	+	+	+	+	+	+	-	-	+
COG0098	Ribosomal protein S5	+	+	-	+	+	+	+	-	+
COG0099	Ribosomal protein S13	+	+	+	+	+	+	+	-	+
COG0100	Ribosomal protein S11	+	+	+	+	+	+	+	-	+
COG0102	Ribosomal protein L13	+	+	+	+	+	+	+	+	+
COG0103	Ribosomal protein S9	+	+	+	+	+	+	+	+	+
COG0124	Histidyl-tRNA synthetase	+	+	+	+	+	+	+	+	+
COG0130	Pseudouridine synthase	+	+	+	+	+	+	+	+	+
COG0143	Methionyl-tRNA synthetase	+	+	+	+	+	+	+	+	+
COG0164	Ribonuclease HII	+	+	+	+	+	+	+	-	+
COG0177	Predicted EndoIII-related endonuclease	+	+	+	+	+	+	+	+	+
COG0180	Tryptophanyl-tRNA synthetase	+	+	+	+	+	+	+	+	+
COG0185	Ribosomal protein S19	+	+	-	+	+	+	+	-	-
COG0186	Ribosomal protein S17	+	+	+	+	+	+	-	-	-
COG0197	Ribosomal protein L16/L10E	+	+	+	+	+	-	+	+	-

Function id	Function name	J07HQW1	J07HQW2	J07HQX50	J07HN4	J07HN6	J07HR59	J07HX64	J07HX5	J07HB67
COG0200	Ribosomal protein L15	+	+	+	+	+	+	+	-	+
COG0201	Preprotein translocase subunit SecY	+	+	+	+	+	+	+	-	+
COG0250	Transcription antiterminator	+	+	-	+	+	+	+	+	+
COG0256	Ribosomal protein L18	+	+	-	+	+	+	+	-	+
COG0361	Translation initiation factor 1 (IF-1)	+	+	+	+	+	+	+	+	+
COG0441	Threonyl-tRNA synthetase	+	+	+	+	+	+	+	+	+
COG0455	ATPases involved in chromosome partitioning	+	-	+	+	+	-	+	-	+
COG0459	Chaperonin GroEL (HSP60 family)	+	+	+	+	+	+	+	+	+
COG0468	RecA/RadA recombinase	+	+	+	+	+	+	+	+	+
COG0480	Translation elongation factors (GTPases)	+	+	+	+	+	+	+	-	+
COG0495	Leucyl-tRNA synthetase	+	+	+	+	+	+	+	+	+
COG0522	Ribosomal protein S4 and related proteins	+	+	+	+	+	+	+	-	+
COG0525	Valyl-tRNA synthetase	+	+	+	+	+	+	+	+	+
COG0532	Translation initiation factor 2 (IF-2; GTPase)	+	+	+	+	+	-	-	+	+
