# Supplementary figures and images for: Assembly-Driven Community Genomics of a Hypersaline Microbial Ecosystem
Source: PLoS One. 2013 Apr 18;8(4):e61692. doi: 10.1371/journal.pone.0061692 (PMC3630111; doi:10.1371/journal.pone.0061692)

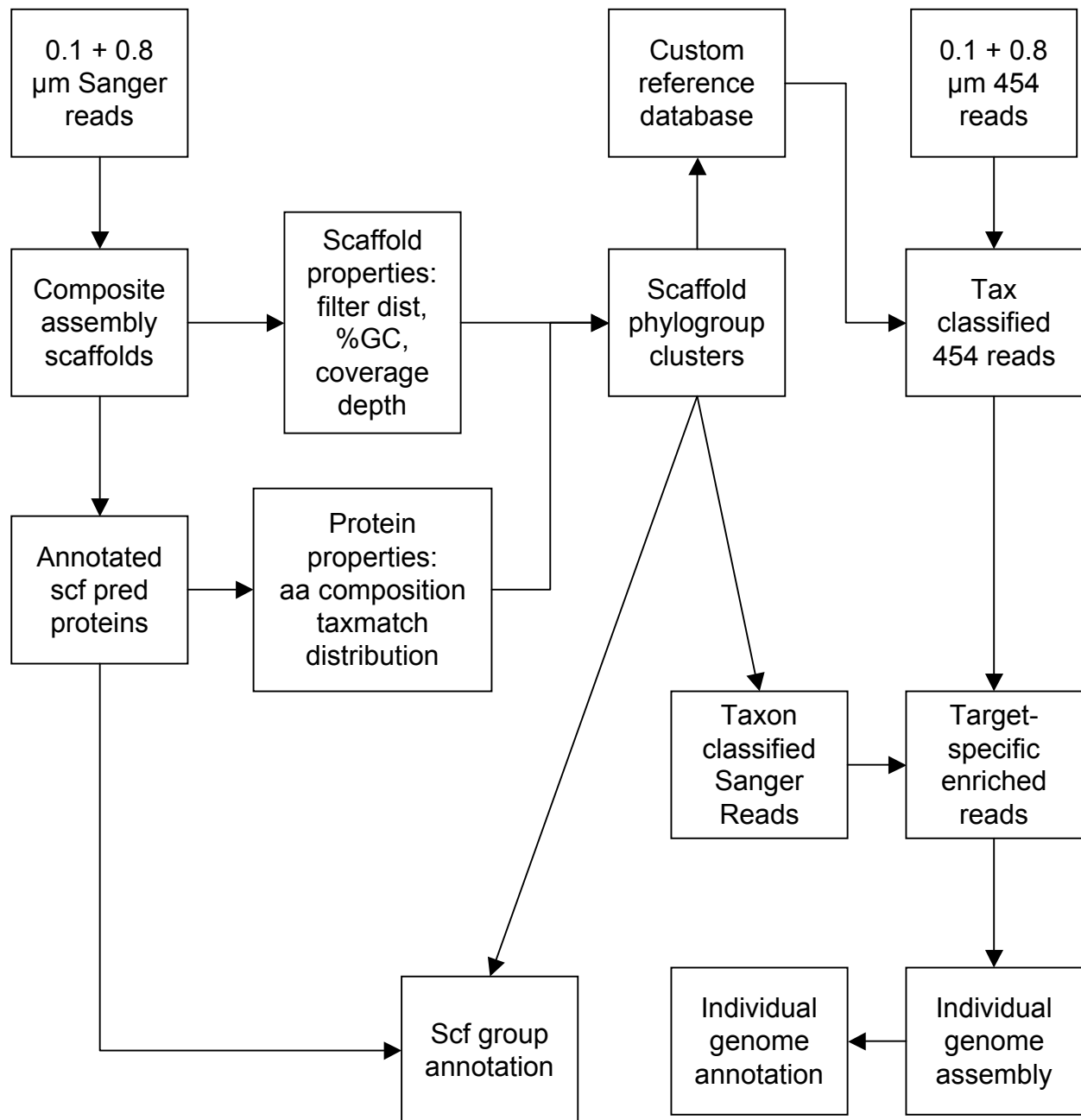

**Supporting Figure S1.** Bioinformatic analysis pipeline.

Supplement: Figure S1 — Bioinformatic Analysis Pipeline. (PDF) [file pone.0061692.s007.pdf]
