# Supplementary material for: Assembly-Driven Community Genomics of a Hypersaline Microbial Ecosystem
Source: PLoS One. 2013 Apr 18;8(4):e61692. doi: 10.1371/journal.pone.0061692 (PMC3630111; doi:10.1371/journal.pone.0061692)
Supplement: Figure S2 — Phylogenetic trees showing abundance of clustered archaeal 16S rRNA sequences from (A) unassembled reads and (B) PCR-amplified clone libraries. A maximum likelihood archaeal reference tree was constructed using FastTree [1], based on full-length 16S genes from isolate genomes and environmental clones from Genbank nt, supplemented with sequences obtained from Lake Tyrrell assembled scaffolds (highlighted in yellow). Additional partial 16S rRNA sequences from Lake Tyrrell were inserted into the reference tree using pplacer version v1.1 (model GTR, fig-eval-all) [2] and visualized using Archaeopteryx 0.968 [3]. Part A shows placement of un-amplified raw metagenomic reads containing 16S gene sequences. Part B shows placement of PCR-amplified 16S rRNA clones. Numbers at nodes indicate confidence values estimated by FastTree for the reference tree. Red lines indicate branches where Lake Tyrrell sequences were observed. The thickness of each red line is proportional to the number of Lake Tyrrell sequences associated with that branch, ranging from one in the thinnest line to 74 in the thickest line. (PDF) [file pone.0061692.s008.pdf]

Supporting Figure S2. Phylogenetic trees showing abundance of clustered archaeal 16S rRNA sequences from (A) unassembled reads and (B) PCR-amplified clone libraries. A maximum likelihood archaeal reference tree was constructed using FastTree [1], based on full-length 16S genes from isolate genomes and environmental clones from Genbank nt, supplemented with sequences obtained from Lake Tyrrell assembled scaffolds. Unassembled 16S rRNA sequences from Lake Tyrrell were clustered and inserted into the reference tree using pplacer version v1.1 (model GTR, fig-eval-all) [2] and visualized using Archaeopteryx 0.968 [3]. Part A shows placement of un-amplified Lake Tyrrell raw reads containing 16S gene sequences > 350 nt. Part B shows placement of PCR-amplified 16S rRNA clones (> nt). Numbers at nodes indicate confidence values estimated by FastTree for the reference database. Red lines indicate branches where Lake Tyrrell sequences were observed. The thickness of each red line is proportional to the number of Lake Tyrrell sequences associated with that branch, ranging from one in the thinnest line to 74 in the thickest line.

References

1. Price, M.N., P.S. Dehal, and A.P. Arkin, FastTree 2--approximately maximum-likelihood trees for large alignments. PLoS One, 2010. 5(3): p. e9490.

2. Matsen,F.,A., Kodner,R., Armbrust, E.V. (2010) pplacer:linear time maximum-likelihood and Bayesian phylogenetic placement of sequences on a fixed reference tree. BMC Bioinformatics, 11:538

3. Han M.V. and Zmasek C.M. (2009). phyloXML: XML for evolutionary biology and comparative genomics. BMC Bioinformatics, 10:356.

A

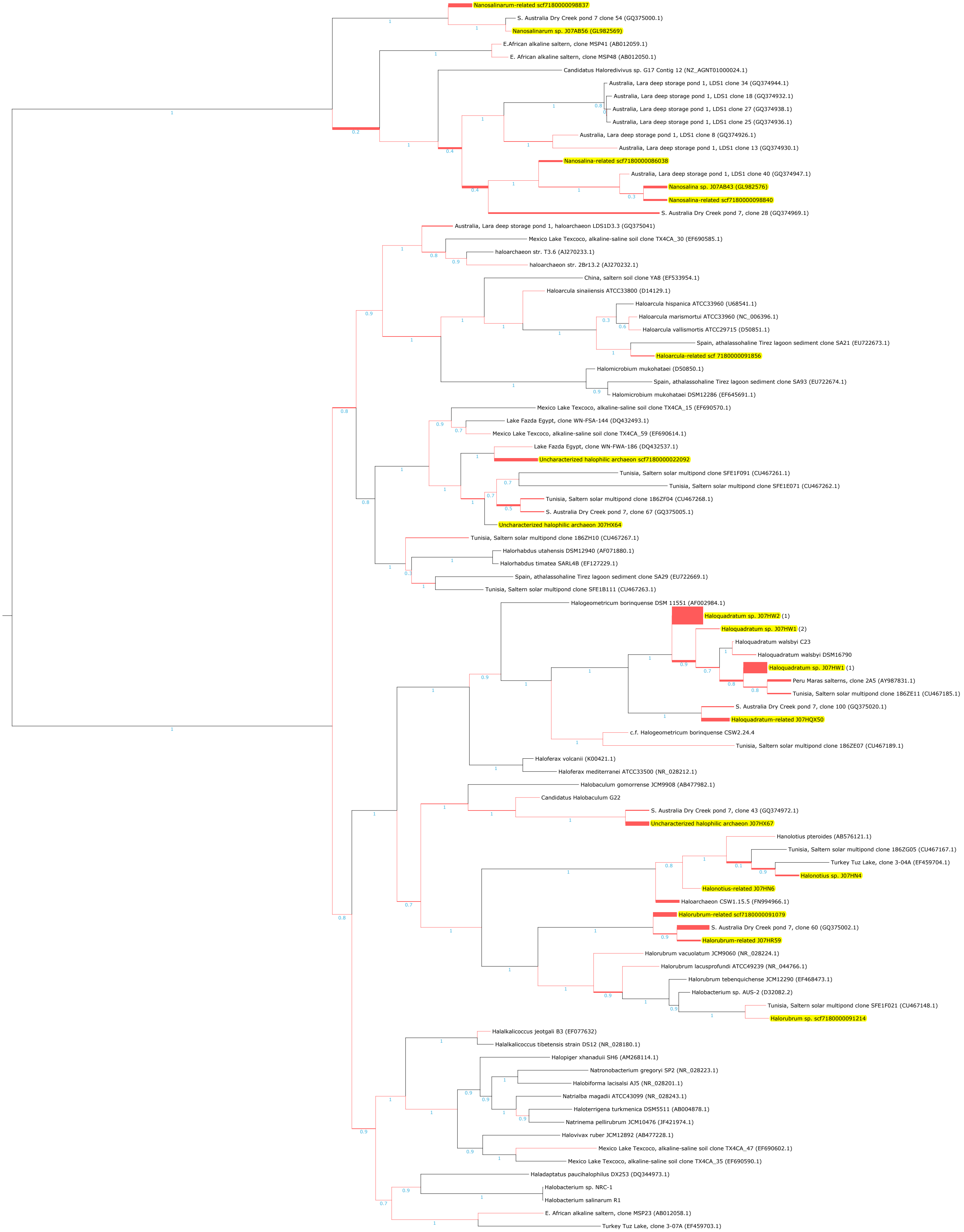
Nanosalinarum-related scf7180000098837
S. Australia Dry Creek pond 7 clone 54 (GQ375000.1)
Nanosalinarum sp. J07AB56 (GL982569)
E.African alkaline saltern, clone MSP41 (AB012059.1)
E. African alkaline saltern, clone MSP48 (AB012050.1)
Candidatus Haloredivivus sp. G17 Contig 12 (NZ_AGNT01000024.1)
Australia, Lara deep storage pond 1, LDS1 clone 34 (GQ374944.1)
Australia, Lara deep storage pond 1, LDS1 clone 18 (GQ374932.1)
Australia, Lara deep storage pond 1, LDS1 clone 27 (GQ374938.1)
Australia, Lara deep storage pond 1, LDS1 clone 25 (GQ374936.1)
Australia, Lara deep storage pond 1, LDS1 clone 8 (GQ374926.1)
Australia, Lara deep storage pond 1, LDS1 clone 13 (GQ374930.1)
Nanosalina-related scf7180000086038
Australia, Lara deep storage pond 1, LDS1 clone 40 (GQ374947.1)
Nanosalina sp. J07AB43 (GL982576)
Nanosalina-related scf7180000098840
S. Australia Dry Creek pond 7, clone 28 (GQ374969.1)
Australia, Lara deep storage pond 1, haloarchaeon LDS1D3.3 (GQ375041)
Mexico Lake Texcoco, alkaline-saline soil clone TX4CA_30 (EF690585.1)
haloarchaeon str. T3.6 (AJ270233.1)
haloarchaeon str. 2Br13.2 (AJ270232.1)
China, saltern soil clone YA8 (EF533954.1)
Haloarcula sinaiiensis ATCC33800 (D14129.1)
Haloarcula hispanica ATCC33960 (U68541.1)
Haloarcula marismortui ATCC33960 (NC_006396.1)
Haloarcula vallismortis ATCC29715 (D50851.1)
Spain, athalassohaline Tirez lagoon sediment clone SA21 (EU722673.1)
Haloarcula-related scf 7180000091856
Halomicrobium mukohataei (D50850.1)
Spain, athalassohaline Tirez lagoon sediment clone SA93 (EU722674.1)
Halomicrobium mukohataei DSM12286 (EF645691.1)
Mexico Lake Texcoco, alkaline-saline soil clone TX4CA_15 (EF690570.1)
Lake Fazda Egypt, clone WN-FSA-144 (DQ432493.1)
Mexico Lake Texcoco, alkaline-saline soil clone TX4CA_59 (EF690614.1)
Lake Fazda Egypt, clone WN-FWA-186 (DQ432537.1)
Uncharacterized halophilic archaeon scf7180000022092
Tunisia, Saltern solar multipond clone SFE1F091 (CU467261.1)
Tunisia, Saltern solar multipond clone SFE1E071 (CU467262.1)
Tunisia, Saltern solar multipond clone 186ZF04 (CU467268.1)
S. Australia Dry Creek pond 7, clone 67 (GQ375005.1)
Uncharacterized halophilic archaeon J07HX64
Tunisia, Saltern solar multipond clone 186ZH10 (CU467267.1)
Halorhabdus utahensis DSM12940 (AF071880.1)
Halorhabdus timatea SARL4B (EF127229.1)
Spain, athalassohaline Tirez lagoon sediment clone SA29 (EU722669.1)
Tunisia, Saltern solar multipond clone SFE1B111 (CU467263.1)
Halogeometricum borinquense DSM 11551 (AF002984.1)
Haloquadratum sp. J07HW2 (1)
Haloquadratum sp. J07HW1 (2)
Haloquadratum walsbyi C23
Haloquadratum walsbyi DSM16790
Haloquadratum sp. J07HW1 (1)
Peru Maras salterns, clone 2A5 (AY987831.1)
Tunisia, Saltern solar multipond clone 186ZE11 (CU467185.1)
S. Australia Dry Creek pond 7, clone 100 (GQ375020.1)
Haloquadratum-related J07HQX50
c.f. Halogeometricum borinquense CSW2.24.4
Tunisia, Saltern solar multipond clone 186ZE07 (CU467189.1)
Haloferax volcanii (K00421.1)
Haloferax mediterranei ATCC33500 (NR_028212.1)
Halobaculum gomorrense JCM9908 (AB477982.1)
Candidatus Halobaculum G22
S. Australia Dry Creek pond 7, clone 43 (GQ374972.1)
Uncharacterized halophilic archaeon J07HX67
Hanolotius pteroides (AB576121.1)
Tunisia, Saltern solar multipond clone 186ZG05 (CU467167.1)
Turkey Tuz Lake, clone 3-04A (EF459704.1)
Halonotius sp. J07HN4
Halonotius-related J07HN6
Haloarchaeon CSW1.15.5 (FN994966.1)
Halorubrum-related scf7180000091079
S. Australia Dry Creek pond 7, clone 60 (GQ375002.1)
Halorubrum-related J07HR59
Halorubrum vacuolatum JCM9060 (NR_028224.1)
Halorubrum lacusprofundi ATCC49239 (NR_044766.1)
Halorubrum tebenquichense JCM12290 (EF468473.1)
Halobacterium sp. AUS-2 (D32082.2)
Tunisia, Saltern solar multipond clone SFE1F021 (CU467148.1)
Halorubrum sp. scf7180000091214
Halalkalicoccus jeotgali B3 (EF077632)
Halalkalicoccus tibetensis strain DS12 (NR_028180.1)
Halopiger xhanaduii SH6 (AM268114.1)
Natronobacterium gregoryi SP2 (NR_028223.1)
Halobiforma lacisalsi AJ5 (NR_028201.1)
Natrialba magadii ATCC43099 (NR_028243.1)
Haloterrigena turkmenica DSM5511 (AB004878.1)
Natrinema pellirubrum JCM10476 (JF421974.1)
Halovivax ruber JCM12892 (AB477228.1)
Mexico Lake Texcoco, alkaline-saline soil clone TX4CA_47 (EF690602.1)
Mexico Lake Texcoco, alkaline-saline soil clone TX4CA_35 (EF690590.1)
Haladaptatus paucihalophilus DX253 (DQ344973.1)
Halobacterium sp. NRC-1
Halobacterium salinarum R1
E. African alkaline saltern, clone MSP23 (AB012058.1)
Turkey Tuz Lake, clone 3-07A (EF459703.1)

B

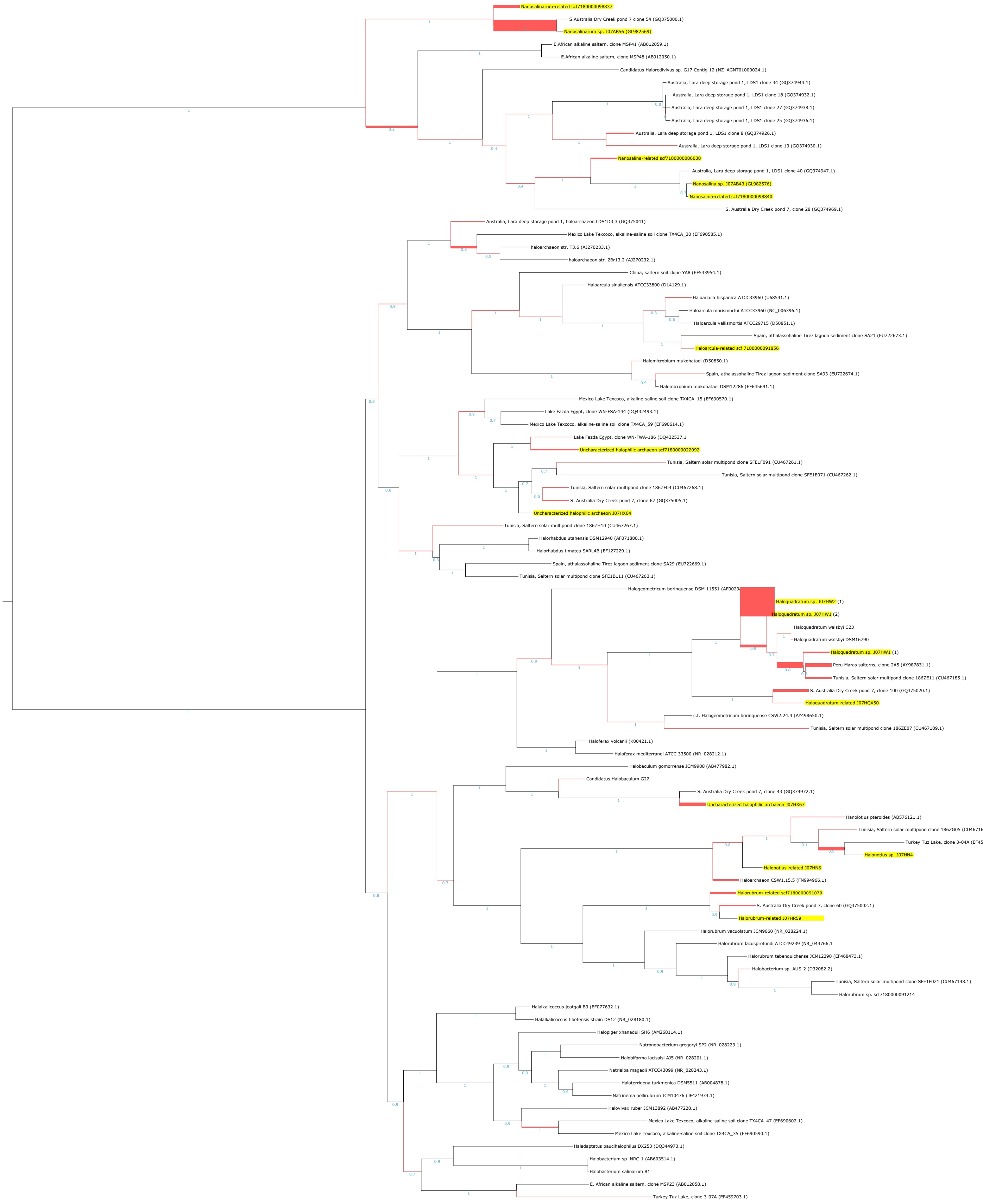

Nanosalinarum-related scf7180000098837
S.Australia Dry Creek pond 7 clone 54 (GQ375000.1)
Nanosalinarum sp. J07AB56 (GL982569)
E.African alkaline saltern, clone MSP41 (AB012059.1)
E.African alkaline saltern, clone MSP48 (AB012050.1)
Candidatus Haloredivivus sp. G17 Contig 12 (NZ_AGNT01000024.1)
Australia, Lara deep storage pond 1, LDS1 clone 34 (GQ374944.1)
Australia, Lara deep storage pond 1, LDS1 clone 18 (GQ374932.1)
Australia, Lara deep storage pond 1, LDS1 clone 27 (GQ374938.1)
Australia, Lara deep storage pond 1, LDS1 clone 25 (GQ374936.1)
Australia, Lara deep storage pond 1, LDS1 clone 8 (GQ374926.1)
Australia, Lara deep storage pond 1, LDS1 clone 13 (GQ374930.1)
Nanosalina-related scf7180000086038
Australia, Lara deep storage pond 1, LDS1 clone 40 (GQ374947.1)
Nanosalina sp. J07AB43 (GL982576)
Nanosalina-related scf7180000098840
S. Australia Dry Creek pond 7, clone 28 (GQ374969.1)
Australia, Lara deep storage pond 1, haloarchaeon LDS1D3.3 (GQ375041)
Mexico Lake Texcoco, alkaline-saline soil clone TX4CA_30 (EF690585.1)
haloarchaeon str. T3.6 (AJ270233.1)
haloarchaeon str. 2Br13.2 (AJ270232.1)
China, saltern soil clone YA8 (EF533954.1)
Haloarcula sinaiiensis ATCC33800 (D14129.1)
Haloarcula hispanica ATCC33960 (U68541.1)
Haloarcula marismortui ATCC33960 (NC_006396.1)
Haloarcula vallismortis ATCC29715 (D50851.1)
Spain, athalassohaline Tirez lagoon sediment clone SA21 (EU722673.1)
Haloarcula-related scf 7180000091856
Halomicrobium mukohataei (D50850.1)
Spain, athalassohaline Tirez lagoon sediment clone SA93 (EU722674.1)
Halomicrobium mukohataei DSM12286 (EF645691.1)
Mexico Lake Texcoco, alkaline-saline soil clone TX4CA_15 (EF690570.1)
Lake Fazda Egypt, clone WN-FSA-144 (DQ432493.1)
Mexico Lake Texcoco, alkaline-saline soil clone TX4CA_59 (EF690614.1)
Lake Fazda Egypt, clone WN-FWA-186 (DQ432537.1
Uncharacterized halophilic archaeon scf7180000022092
Tunisia, Saltern solar multipond clone SFE1F091 (CU467261.1)
Tunisia, Saltern solar multipond clone SFE1E071 (CU467262.1)
Tunisia, Saltern solar multipond clone 186ZF04 (CU467268.1)
S. Australia Dry Creek pond 7, clone 67 (GQ375005.1)
Uncharacterized halophilic archaeon J07HX64
Tunisia, Saltern solar multipond clone 186ZH10 (CU467267.1)
Halorhabdus utahensis DSM12940 (AF071880.1)
Halorhabdus timatea SARL4B (EF127229.1)
Spain, athalassohaline Tirez lagoon sediment clone SA29 (EU722669.1)
Tunisia, Saltern solar multipond clone SFE1B111 (CU467263.1)
Halogeometricum borinquense DSM 11551 (AF0029
Haloquadratum sp. J07HW2 (1)
Haloquadratum sp. J07HW1 (2)
Haloquadratum walsbyi C23
Haloquadratum walsbyi DSM16790
Haloquadratum sp. J07HW1 (1)
Peru Maras salterns, clone 2A5 (AY987831.1)
Tunisia, Saltern solar multipond clone 186ZE11 (CU467185.1)
S. Australia Dry Creek pond 7, clone 100 (GQ375020.1)
Haloquadratum-related J07HQX50
c.f. Halogeometricum borinquense CSW2.24.4 (AY498650.1)
Tunisia, Saltern solar multipond clone 186ZE07 (CU467189.1)
Haloferax volcanii (K00421.1)
Haloferax mediterranei ATCC 33500 (NR_028212.1)
Halobaculum gomorrense JCM9908 (AB477982.1)
Candidatus Halobaculum G22
S. Australia Dry Creek pond 7, clone 43 (GQ374972.1)
Uncharacterized halophilic archaeon J07HX67
Hanolotius pteroides (AB576121.1)
Tunisia, Saltern solar multipond clone 186ZG05 (CU467167.1)
Turkey Tuz Lake, clone 3-04A (EF459704.1)
Halonotius sp. J07HN4
Halonotius-related J07HN6
Haloarchaeon CSW1.15.5 (FN994966.1)
Halorubrum-related scf7180000091079
S. Australia Dry Creek pond 7, clone 60 (GQ375002.1)
Halorubrum-related J07HR59
Halorubrum vacuolatum JCM9060 (NR_028224.1)
Halorubrum lacusprofundi ATCC49239 (NR_044766.1
Halorubrum tebenquichense JCM12290 (EF468473.1)
Halobacterium sp. AUS-2 (D32082.2)
Tunisia, Saltern solar multipond clone SFE1F021 (CU467148.1)
Halorubrum sp. scf7180000091214
Halalkalicoccus jeotgali B3 (EF077632.1)
Halalkalicoccus tibetensis strain DS12 (NR_028180.1)
Halopiger xhanaduii SH6 (AM268114.1)
Natronobacterium gregoryi SP2 (NR_028223.1)
Halobiforma lacisalsi AJ5 (NR_028201.1)
Natrialba magadii ATCC43099 (NR_028243.1)
Haloterrigena turkmenica DSM5511 (AB004878.1)
Natrinema pellirubrum JCM10476 (JF421974.1)
Halovivax ruber JCM13892 (AB477228.1)
Mexico Lake Texcoco, alkaline-saline soil clone TX4CA_47 (EF690602.1)
Mexico Lake Texcoco, alkaline-saline soil clone TX4CA_35 (EF690590.1)
Haladaptatus paucihalophilus DX253 (DQ344973.1)
Halobacterium sp. NRC-1 (AB603514.1)
Halobacterium salinarum R1
E. African alkaline saltern, clone MSP23 (AB012058.1)
Turkey Tuz Lake, clone 3-07A (EF459703.1)
