# Supplementary material for: Assembly-Driven Community Genomics of a Hypersaline Microbial Ecosystem
Source: PLoS One. 2013 Apr 18;8(4):e61692. doi: 10.1371/journal.pone.0061692 (PMC3630111; doi:10.1371/journal.pone.0061692)
Supplement: Figure S3 — Non-metric multidimensional scaling plot illustrating distinctive scaffold groups. Scaffolds >5 Kb from the composite Sanger assembly were subjected to non-metric multidimensional scaling analysis using Primer 1.6, with Euclidean distance, 25 random starts, Krustal fit scheme 1, and minimum stress value 0.01 for the 13 parameters shown in Table S5. Axes shown are arbitrary units of composite clustering, although the X axis appears to be dominated by nucleotide percent G+C. Scaffolds associated with major taxonomic groups are highlighted with colored symbols. Small grey dots indicate scaffolds that could not be unambiguously classified into major groups. (PDF) [file pone.0061692.s009.pdf]

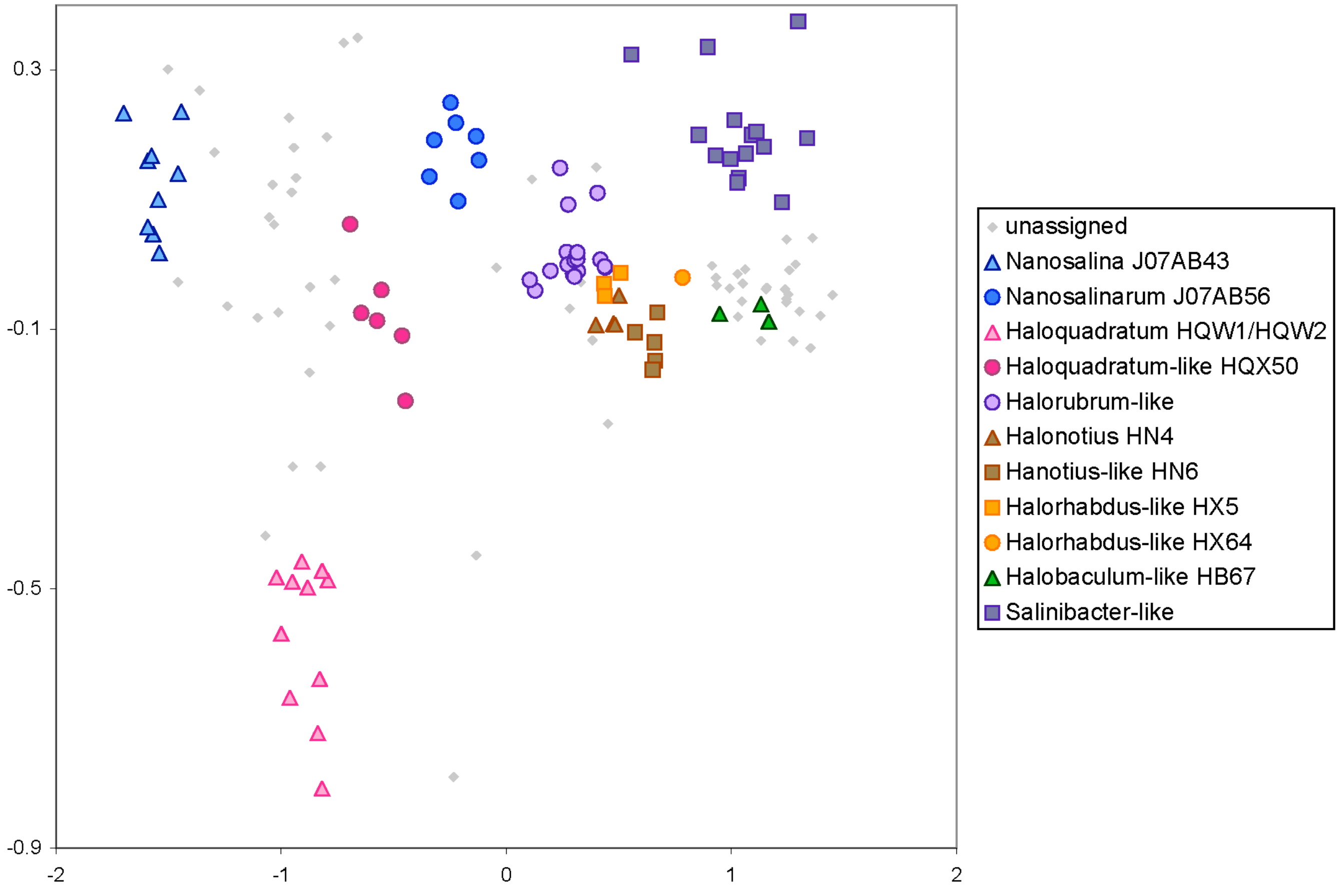

Supplementary Figure S3. Non-metric multidimensional scaling plot illustrating distinctive scaffold groups. Scaffolds > 5Kb from the composite Sanger assembly were subjected to non-metric multidimensional scaling analysis using Primer 1.6, with Euclidean distance, 25 random starts, Krustal fit scheme 1, and minimum stress value 0.01 for the 13 parameters shown in Supplementary Table S5. Axes shown are arbitrary units of composite clustering, although the X axis appears to be dominated by nucleotide percent G+C. Scaffolds associated with major taxonomic groups are highlighted with colored symbols. Small grey dots indicate scaffolds that could not be unambiguously classified into major groups.
