# Supplementary material for: Assembly-Driven Community Genomics of a Hypersaline Microbial Ecosystem
Source: PLoS One. 2013 Apr 18;8(4):e61692. doi: 10.1371/journal.pone.0061692 (PMC3630111; doi:10.1371/journal.pone.0061692)
Supplement: Figure S4 — Rank abundance of assembled microbial populations based on depth of coverage. (PDF) [file pone.0061692.s010.pdf]

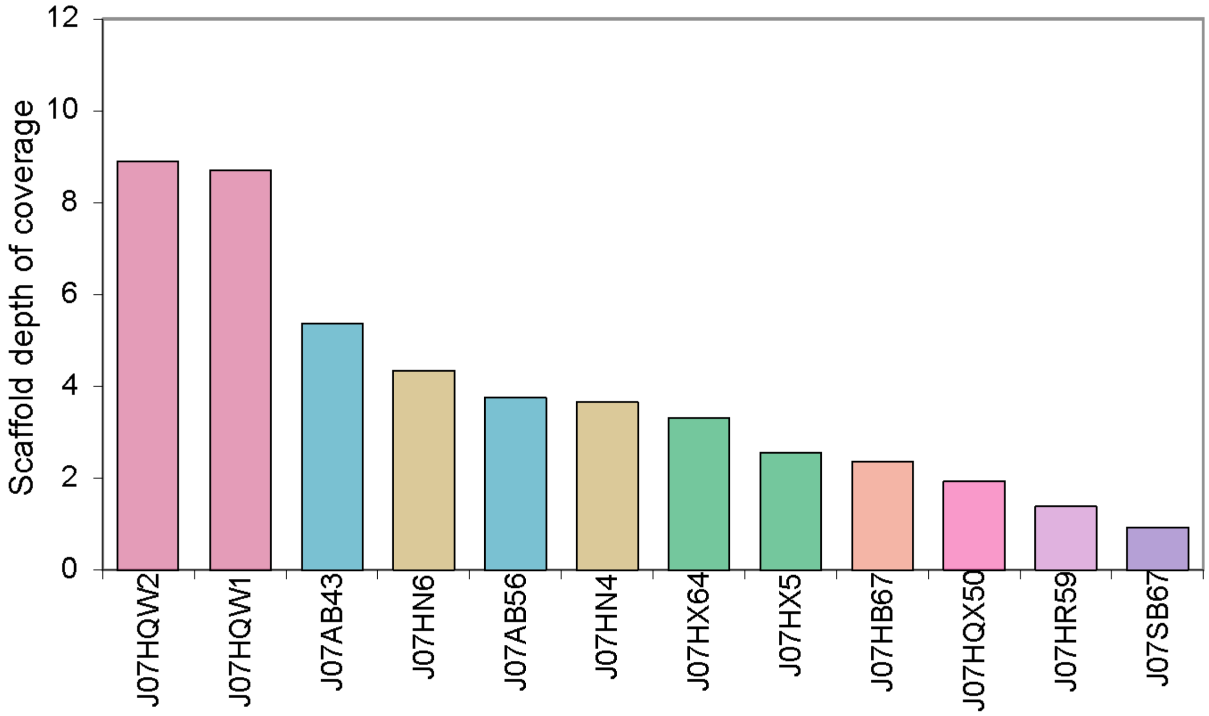

Supplementary Figure S4. Rank abundance of assembled microbial populations based on depth of coverage.
